# Targeted Isolation of Xenicane Diterpenoids From Taiwanese Soft Coral *Asterospicularia laurae*

**DOI:** 10.3390/md19030123

**Published:** 2021-02-25

**Authors:** Yu-Chi Lin, Yi-Jen Chen, Shu-Rong Chen, Wan-Ju Lien, Hsueh-Wei Chang, Yu-Liang Yang, Chia-Ching Liaw, Jui-Hsin Su, Ching-Yeu Chen, Yuan-Bin Cheng

**Affiliations:** 1 Department of Marine Biotechnology and Resources, National Sun Yat-sen University, Kaohsiung 804351, Taiwan; m8952612@hotmail.com (Y.-C.L.); ylyang@gate.sinica.edu.tw (Y.-L.Y.); 2 Department of Fragrance and Cosmetic Science, College of Pharmacy, Kaohsiung Medical University, Kaohsiung 807378, Taiwan; mermaid190111@gmail.com; 3 Graduate Institute of Natural Products, Center for Natural Product Research and Development, College of Pharmacy, Kaohsiung Medical University, Kaohsiung 807378, Taiwan; highshorter@hotmail.com; 4 Department of Biomedical Science and Environmental Biology, PhD Program in Life Science, College of Life Science, Kaohsiung Medical University, Kaohsiung 80708, Taiwan; rfsl.lien@gmail.com (W.-J.L.); changhw@kmu.edu.tw (H.-W.C.); 5 Center for Cancer Research, Kaohsiung Medical University, Kaohsiung 80708, Taiwan; 6 Cancer Center, Kaohsiung Medical University Hospital, Kaohsiung 80708, Taiwan; 7 Department of Medical Research, Kaohsiung Medical University Hospital, Kaohsiung 80708, Taiwan; 8 Agricultural Biotechnology Research Center, Academia Sinica, Taipei 115, Taiwan; 9 Division of Chinese Materia Medica Development, National Research Institute of Chinese Medicine, Taipei 11221, Taiwan; liawcc@nricm.edu.tw; 10 Department of Biochemical Science and Technology, National Chiayi University, Chiayi 60004, Taiwan; 11 Graduate Institute of Marine Biology, National Dong Hwa University, Pingtung 944401, Taiwan; x2219@nmmba.gov.tw; 12 Department of Physical Therapy, Tzu-Hui Institute of Technology, Pingtung 92641, Taiwan

**Keywords:** *Asterospicularia laurae*, GNPS molecular networking, xenicane, cytotoxic

## Abstract

Application of LC-MS/MS-based molecular networking indicated the ethanol extract of octocoral *Asterospicularia laurae* is a potential source for the discovery of new xenicane derivatives. A natural product investigation of this soft coral resulted in the isolation of four new xenicane diterpenoids, asterolaurins O–R (**1**–**4**), together with six known compounds, xeniolide-A (**5**), isoxeniolide-A (**6**), xeniolide-B (**7**), 7,8-epoxyxeniolide-B (**8**), 7,8-oxido-isoxeniolide-A (**9**), and 9-hydroxyxeniolide-F (**10**). The structures of isolated compounds were characterized by employing spectroscopic analyses, including 2D-NMR (COSY, HMQC, HMBC, and NOESY) and high-resolution electrospray ionization mass spectrometry (HRESIMS). Asterolaurin O is the first case of brominated tricarbocyclic type floridicin in the family Xeniidae. Concerning bioactivity, the cytotoxic activity of those isolates was evaluated. As a result, compounds **1** and **2** demonstrated a selective cytotoxic effect against the MCF-7 cell line at IC_50_ of 14.7 and 25.1 μM, respectively.

## 1. Introduction

Marine organisms such as sponges, soft corals, tunicates, and alga were regarded as plentiful sources of bioactive molecules, and many marine natural products or their derivatives have been used as drug candidates. Over the past 40 years, nearly 59% of antitumor agents have come from small-molecule natural products or inspired from natural sources [1]. So far, natural product investigations of *Asterospicularia* sp. resulted in a new pentahydroxylated sterol named 24*ξ*-Methyl-5*α*-cholestane-3*β*,5,6*β*,22*R*,24-pentol 6-acetate together with 14 new xenicane-type diterpenoids (13-*epi*-9-deacetoxyxenicin and asterolaurins A–M) [2,3,4,5,6,7]. Among those isolates, 13-*epi*-9-deacetoxyxenicin exhibited strong cytotoxicity against P388D_1_ mouse lymphoma cells with an IC_50_ of 2.17 μM [3], asterolaurin A exhibited moderate cytotoxicity against HepG2 cells with an IC_50_ of 8.9 µM, asterolaurin D showed inhibition of elastase release and superoxide anion generation with IC_50_ values of 18.7 and 23.6 µM, respectively [4], asterolaurin L showed moderate cytotoxic activity against HEp-2, Daoy, MCF-7, and WiDr tumor cell lines, with ED_50_ values of 11.8, 17.8, 11.7, and 17.4 μg/mL, respectively [5]. Moreover, 13-*epi*-9-desacetylxenicin, first isolated from *Xenia Novae-Britanniae*, also yielded from *A. laurae*, demonstrated significantly cytotoxic against Molt 4 (human T lymphoblast; acute lymphoblastic leukemia), K562 (human blood chronic myelogenous leukemia), Sup-T1 (Human T cell lymphoblastic lymphoma), and U937 (Human Caucasian histiocytic lymphoma) cells with IC_50_ values of 1.30, 1.19, 3.17, 2.45 μM, respectively [7]. LC-MS/MS-based metabolite profiling has gradually become the mainstream of modern natural product investigation. This method provides a quick and visible spectrum for natural product de-replication [8,9] and targeted isolation [10]. As an assistant of this approach, *A. laurae*., collected in Orchid Island, Taiwan, were evaluated, and it demonstrated an abundance of xenicane-type diterpenes. As stated above, xenicane-type diterpenes could be potential sources of new antitumor agents; therefore, our continuing marine natural product investigation of bioactivity substances focuses on it. This article reports the isolation, structure determination, and bioactivity evaluation of the marine metabolites isolated from *A. laurae*.

## 2. Results and Discussion

The ethanol extract of *A. laurae* was partitioned between EtOAc and H_2_O to obtain an EtOAc-soluble layer. This layer was further partitioned between hexanes and 75% MeOH_(aq)_ to remove the low polarity metabolites. The 75% MeOH layer was analyzed by the LC-MS/MS (negative ion mode). The MS/MS data were uploaded to the Global Natural Products Social Molecular Networking (GNPS, https://gnps.ucsd.edu/ (accessed on 3 February 2021)) website, and the output data were mapped to create correlated clusters.

**Figure 1:**
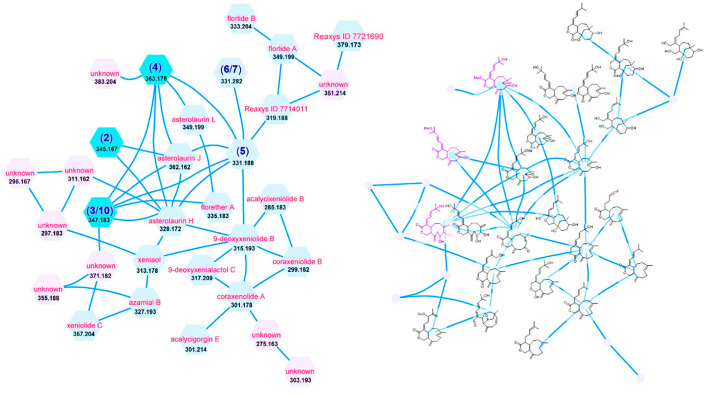
Cluster (left: molecular weight and proposed name; right: proposed chemical structure) of the xenicane-type diterpenes from the extract of *A. laurae*.

A cluster (Figure 1) with molecular weights of nodes between 276 and 384 was found to have the MS/MS fragment peaks of xenicane-type diterpenes (Figure S45), suggesting this group of metabolites could be xenicane-type diterpenes. A de-replication work was subsequently executed by comparing those molecular weights of nodes to known xenicanes. This investigation indicated that *A. laurae* should be a rich sources of new xenicane-type diterpenes.

**Figure 2:**
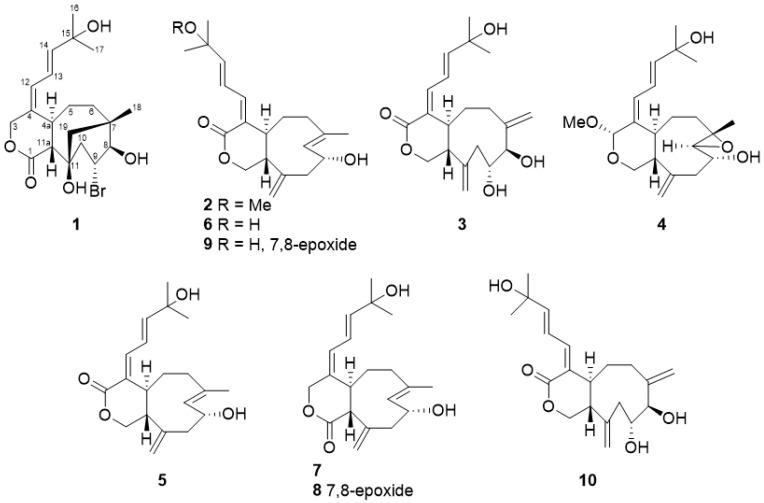
Structures of compounds **1**–**10** isolated from *A. laurae.*

Four new compounds named asterolaurins O–R (**1**–**4**) along with six known xenicane diterpenoids, xeniolide-A (**5**) [11], isoxeniolide-A (**6**) [12], xeniolide-B (**7**) [11], 7,8-epoxyxeniolide-B (**8**) [13], 7,8-oxido-isoxeniolide-A (**9**) [11], and 9-hydroxyxeniolide-F (**10**) [14] (Figure 2) were isolated and purified by successive silica gel, Sephadex LH-20, and semi-preparative normal-phase and reversed-phase high performance liquid chromatography (HPLC) columns. Their structures were further elucidated by spectroscopic data and compared with the relative literature.

**Figure 3:**
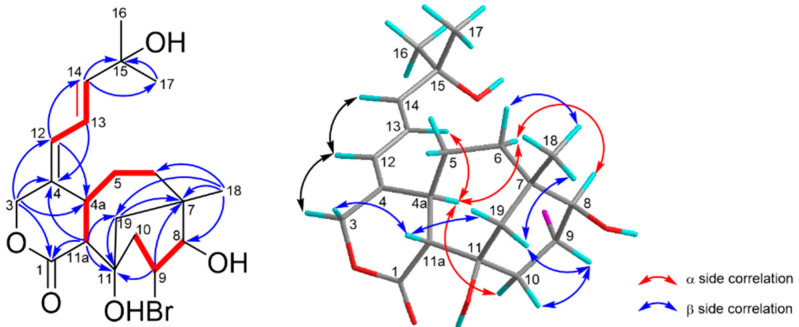
COSY (bold bond), selected HMBC (arrow), and NOESY (left-right arrow) correlations of **1**.

Asterolaurin O (**1**) was obtained as an amorphous, colorless gum. The infrared (IR) data indicated the presence of hydroxy (3424 cm^−1^) and ester carbonyl (1719 cm^−1^) functionalities. The presence of one bromine atom in **1** was apparent from the isotopic pattern in a 1:1 ratio observed for the quasi-molecular ion peaks at 451.11 [M + Na]^+^ and 453.12 [M +Na+ 2]^+^, accompanied by an [M + H]^+^ fragment at m/z 429.04 and 431.11, and its molecular formula was assigned as C_20_H_29_BrO_5_ by high-resolution ESIMS (*m/z* 451.10903 [M + Na]^+^, calculated for C_20_H_29_BrNaO_5_, 451.10906), implying 6 degrees of unsaturation. The ^13^C NMR and distortionless enhancement by polarization transfer (DEPT) spectra showed the presence of four olefinic carbons (δc 122.3 (d), 129.7 (d), 137.0 (c), and 146.2 (d)) and a lactone carbonyl δc 176.9 (s), suggesting that **1** was tricyclic. Detailed inspection of ^1^H and ^13^C NMR spectra of **1** (Table 1) disclosed signals characteristic for the A ring and the side chain were similar to those in florlide A [15], such as two singlet methyl protons (δ_H_ 1.30 x2) on a quaternary carbon (δ_C_ 71.3, C-15) substituted by a hydroxyl group, were assigned to H-16 and H-17. Moreover, the diene resonance due to H-13 (δ_H_ 6.34, dd, *J* = 11.1, 15.3 Hz) was correspondingly coupled to H-12 (δ_H_ 6.15, d, *J* = 11.1 Hz) and H-14 (δ_H_ 5.94, d, *J* = 15.3 Hz). Additionally, the chemical shifts of diene protons combined with the appearance of lactone carbonyl signal, as well as an AB spin system oxymethylene at δ_H_ 4.44 (1H, d, *J*= 12.0 Hz) and 5.06 (1H, br d, *J* = 12.0 Hz) implied that **1** should belong to a xeniolide B type pyran-cyclononane diterpenoid. Analysis of the ^1^H-^1^H COSY and HMBC spectra (Figure 3) corroborated the plane structure of **1**. COSY correlations between two de-shielded protons H-8 (δ_H_ 4.09, d, 5.7; δ_C_ 72.9) and H-9 (δ_H_ 4.37, td, 5.7, 8.7; δ_C_ 75.1), the latter one also correlating to H-10 (δ_H_ 2.22, d, 5.7 and 2.24, d, 8.7; δ_C_ 39.8), were observed. The other spin system for H-11a/H-4a/H-5/H-6 from the COSY spectrum as well as HMBC correlations from Me-18 to C-6, C-7, C-8, and C-19, from isolated AB quartet protons H_2_-19 to C-7, C-11, and C-11a, from H-11a to C-1, C-11, and C-19 had established the bicyclic [4.3.1] ring system in **1**. Two hydroxyl groups were positioned at C-8 and C-11 due to some similar bicyclic [4.3.1] analogues being yielded from *Xenia* species, and possessed the same substitutes [15,16,17,18,19]. Thus, the residue bromine should be attached at the C-9 position to meet the data from NMR and mass, and the gross structure of **1** was identified. The relative stereochemistry of compound **1** was established from NOESY correlations (Figure 3) and by comparison of its spectroscopic data to those of xeniolide analogues. The *E* geometry of the Δ^13^ double bond was established by the large coupling constant observed between H-13 and H-14 (*J* = 15.3 Hz). Furthermore, the geometry of the olefinic bond between C-4 and C-12 was concluded to be *E*, based on a strong NOESY correlation between H-4a (δ_H_ 3.18) and H-13 was observed. The large coupling constant (*J* = 12.0 Hz) between H-4a and H-11a allowed us to assume H-4a was *α*-orientation, whereas H-11a was *β*-orientation. The NOESY correlations of H-19_A_/H-11a/H-19*β*/Me-18 revealed H_2_-19 and Me-18 were both on the *β*-side of **1**. Based on the above results, we could deduce that the stereochemistry of ring junctions (C-7 and C-11) in the bicyclic [4.3.1] scaffold of **1** were the same with those of floridicins [17]. The NOESY correlations of H-10*α*/H-4a/H-6*α*/H-8 revealed those protons were on the *α*-side of **1**. On the contrary, the NOESY correlations of H-6*β*/Me-18/H-19*β*/H-9/H-10*β* revealed that those protons were on the *β*-side of **1**. Therefore, the structure of **1** (asterolaurin O) was assigned as 9*α*-bromo-florlide A on the basis of the above results. This structure represents the first case of brominated tricarbocyclic floridicin among the plethora of diterpenoid compounds already reported from corals.

**Figure 4:**
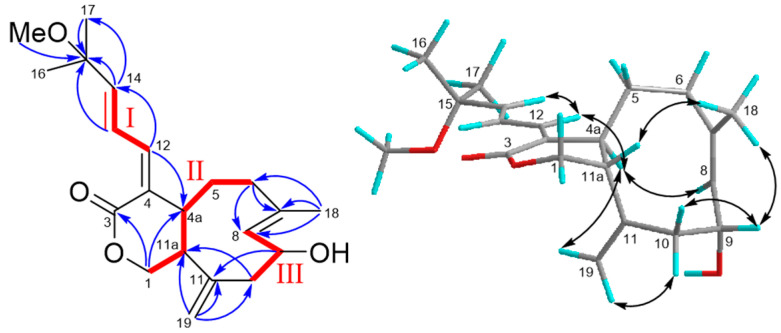
COSY (bold bond), selected HMBC (arrow), and NOESY (left-right arrow) correlations of **2**.

Asterolaurin P (**2**) was obtained as a pale yellowish amorphous gum with a molecular formula of C_21_H_30_O_4_ with 7 indices of hydrogen deficiency, as established based on its ^13^C NMR data and an HRESIMS pseudo-molecular ion peak at *m/z* 369.20379 [M + Na]^+^ (calcd for 369.20363). The IR spectrum indicated absorption bands due to hydroxyl (3454 cm^−1^) and ester carbonyl (1727 cm^−1^) functionalities, whereas the UV (λ_max_ 237 and 215 nm) also supported a conjugated diene system. The structure of **1** was completely identified by a combination of 1D and 2D nuclear magnetic resonance experiments. The carbon resonances at δ_C_ 126.6 (CH), 131.8 (CH), 133.2 (qC),134.6 (qC), 137.3 (CH), and 146.4 (CH) in the ^13^C NMR and DEPT spectra (Table 1) suggested the presence of three double bonds, and the quaternary carbon signal at δ_C_ 149.5 along with the methylene olefinic carbon signal at δ_C_ 115.3 indicated the presence of an *exo*-methylene double bond. Moreover, an ester δ_C_ 171.4 (qC) was also observed that implied that **2** was a bicyclic compound. The ^1^H NMR spectrum (Table 1) confirmed the presence of an *exo*-methylene double bond by two singlet signals at δ_H_ 4.95 and 5.06. Three spin systems (**I**-**III**, Figure 1) were deduced from combined ^1^H-^1^H COSY (Figure 4) and HSQC spectra of **1**. Fragment **I** consisted of a sequence of three double bond methines, and fragment **II** started from an oxymethylene (δ_H_ 3.63, 4.11) and ended with the relative deshielding methylene (δ_H_ 2.19) as well as fragment **III**, which included a carbinolic proton (δ_H_ 4.72; δ_C_ 67.9) and was correlated with the fourth double bond methine (δ_H_ 5.26; δ_C_ 131.8) and with allylic methylene (δ_H_ 2.34 and 2.50). These subunits were connected through key HMBC correlations (Figure 4) of H-1 (δ_H_ 3.63, 4.11) with C-3 (δ_C_ 171.4) and C-4a (δ_C_ 52.0), of H-12 (δ_H_ 6.53) with C-4a, of protons H-13 (δ_H_ 6.76), H-14 (δ_H_ 5.98), Me-16 (δ_H_ 1.30), and methoxy (δ_H_ 3.18) with C-15 (δ_C_ 76.5), of Me-18 (δ_H_ 1.70) with C-6 (δ_C_ 40.9), C-7(δ_C_ 133.2), and C-8 (δ_C_ 131.8), as well as of exomethylene protons (δ_H_ 4.95, 5.06) with C-10 (δ_C_ 46.2), C-11(δ_C_ 49.5), and C-11a (δ_H_ 51.1). Based on the above results, the gross structure of asterolaurin P could be constructed. The coupling constant (*J* = 11.0 Hz) between H-4a and H-11a suggested a *trans* ring junction, which implied that H-4a was *α*-oriented. The *Z* geometry of the Δ^4,12^ double bond was deduced on the basis of the NOESY (Figure 4) cross-peaks H-12/H-4a, and the chemical shift of C-4a in **1** was shifted -9.8 ppm, compared with its Δ^4,12^ *E* isomer, due to an γ effect of C-13 [7]. Additionally, the chemical shift of H-13 at δ_H_ 6.76, which is downfield-shifted to the corresponding *E* isomer (δ_H_ 6.40) due to an anisotropic effect, occurred with the carbonyl group. Moreover, the *E* geometry of the Δ^13^ double bond was established by the large coupling constant observed between H-13 and H-14 (*J* = 15.7 Hz). On the other hand, the Δ^7^ double bond could be determined as an *E* configuration according to the ^13^C chemical shift of Me-18, which was 17.3 rather than at 22-25 for *Z* configuration [20]. The large coupling constant (*J* = 11.0 Hz) between H-4a and H-11a suggested a *trans*-juncture of the two rings, which implied that H-4a was *α*-oriented. The NOESY correlations of H-4a with H-8, which presented quasi-axial on the *α*-face, and on the other hand, of H-11a with Me-18, exhibited a quasi-axial on the *β*-face as well as Me-18 and also showed correlation with H-9 revealed an *α*-orientation of the hydroxyl group at the C-9 position. Therefore, the structure of asterolaurin P was assigned as **2** based on the above results.

**Figure 5:**
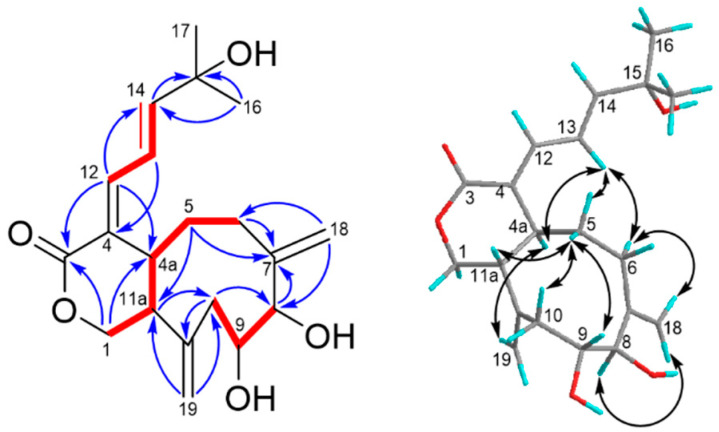
COSY (bold bond), selected HMBC (arrow), and NOESY (left-right arrow) correlations of **3**.

The molecular formula of C_20_H_28_O_5_ was deduced for asterolaurin Q (**3**) from its HRESIMS data, being consistent with 7 indices of hydrogen deficiency. The IR absorptions at 3420 and 1704 cm^−1^ indicated the presence of hydroxy and ester carbonyl groups, respectively. The NMR spectral data of **3** revealed a ring A similar to those of **1** because of an AB system of H_2_-1 at δ_H_ 4.09 (dd, 4.1, 11.0) and 3.91 (d, 11.0 Hz) was observed. The diene system, H-13 at δ_H_ 6.57 (dd, *J*= 11.8, 15.1 Hz), was coupled to H-12 (δ_H_ 7.04, d, *J* = 11.8 Hz) and H-14 (δ_H_ 6.35, d, *J* = 15.1 Hz), whereas the downfield shift of H-12 attributed to an anisotropy effect occurred with carbonyl group at C-3 that implied the *E* form configuration of Δ^4,12^ double bond in **3**. In the aided DEPT spectra, ^13^C NMR resonances at δ_C_ 116.5 (CH_2_) and 120.1 (CH_2_) indicated the presence of two exo methylene double bonds, which were confirmed by the observation of four doublet signals at δ_H_ 4.84 (d, 1.8), 5.02 (d, 1.8), 5.10 (d, 1.6), and 5.20 (d, 1.6) in the ^1^H NMR spectrum. Besides, the presence of two oxygenated methines was deduced from the carbon signal at δ_C_ 83.1 and 70.5. corresponded to the proton signal at δ_H_ 3.97 (d, 9.0) and 4.06 (dd, 3.9, 9.0), respectively. The COSY spectrum (Figure 5) showed cross-peaks with signals at H-12/ H-13/ H-14; H-1 (δ_H_ 4.09, 3.91)/ H-11a (δ_H_ 2.46, m)/ H-4a (δ_H_ 3.17)/ H-5 (δ_H_ 1.84, m)/ H-6 (δ_H_ 1.92, m); H-8 (δ_H_ 3.97)/H-9 (δ_H_ 4.06)/ H-10 (δ_H_ 2.48, 2.60). Furthermore, the key HMBC correlations (Figure 5) of both H-1 and H-12 with C-3 (δ_C_ 171.4) and C-4a (δ_C_ 52.0), as well as H-13, H-14, Me-16 and Me-17 with C-15 (δ_C_ 76.5), allowed a δ-valerolactone ring linked with (via C-4) a diene which extended to an oxyquaternary carbon bearing two geminal methyls in **2**. The HMBC correlations of H-6 with C-8, of H-10 with C-11a, of exomethylene protons (δ_H_ 5.10, 5.20, H-18) with C-6 and C-8, and of exomethylene protons (δ_H_ 5.02, 4.84, H-19) with C-10 and C-11a, allowed the construction of a cyclononane ring with two exomethylene functionalities at C-7 and C-11. Considering the molecular formula of **3** as well as the chemical shifts of C-8 (δ_C_ 83.1), C-9 (δ_C_ 70.5), and C-15 (δ_C_ 71.5), three hydroxy groups were attached at the abovementioned positions. Herein, the gross structure of **3** was assigned. The *trans* junction of the two rings was suggested by coupling constant (*J* = 9.2 Hz) between H-4a and H-11a, and the configuration of H-4a could be assumed as *α*-oriented. Additionally, the NOESY correlations (Figure 4) of H-19/H-4a/H-13 and also the correlations of H-5*α*/H-13/H-6*α*/H-18/H-8 revealed those protons were on the same *α* face of the structure. On the other hand, the NOESY cross-peaks of H-11a/H-5*β*/H-9 implied that H-9 was *β*-orientation. Therefore, the structure of asterolaurin P was assigned as **3** on the basis of the above results.

**Figure 6:**
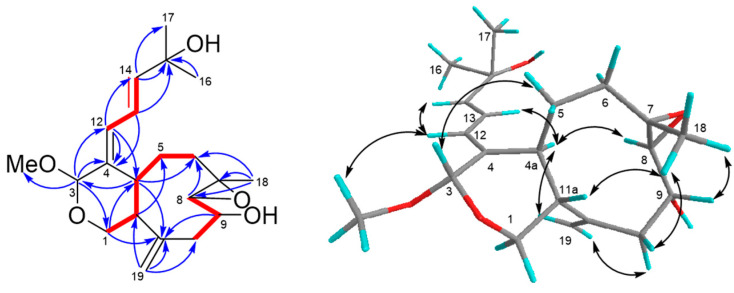
COSY (bold bond), selected HMBC (arrow), and NOESY (left-right arrow) correlations of **4**.

Compound **4** was isolated as an amorphous gum, and its molecular formula was established as C_21_H_32_O_5_ by HREIMS and NMR spectral data. The ^1^H and ^13^C NMR spectra of **4** showed some characteristic signals in the cyclononane skeleton as B ring moiety, similar to those of compounds **8** and **9**. Two singlets at δ_H_ 5.11 and 4.85 corresponding to δ_C_ 144.8 were typical of resonances due to exocyclic methylene protons at C-19. A methyl-bearing *E* trisubstituted epoxide [δ_H_ 1.43 s, 3.00 (d, 8.0); δ_C_ 17.3 q, 59.2 s, 66.9 d], and epoxide proton (H-8) was further shown, and coupled with oxymethine [δ_H_ 3.80, dd (8.0, 7.4), δ_C_ 69.1] it implied a hydroxy group attached at the C-9 position. Moreover, bands for a diene olefinic system at δ_H_ 6.36 (d, 11.3, H-12), 6.44 (dd, 14.9, 11.3, H-13), and 5.84 (d,14.9, H-14) were also observed. COSY and HMBC correlations (Figure 6) supported the structure in which three spin fragments were connected in the aid of key HMBC corrections of Me-18 with C-6, C-7, and C-8, of H-19 with C-10, C-11, and C-11a, of an acetal proton H-3 (δ_H_ 5.17, brs; δ_C_ 99.2) with C-1, C-4, C-12, and methoxyl carbon (δ_C_ 55.1). Therefore, the structure of **4** could be established unambiguously. The 4(12) *E*-configuration and the *E*-geometry for Δ^13^ double bonds were determined by NOESY correlation (Figure 6) between H-13 and H-4a, and the coupling constant between H-13 and H-14 (14.9), respectively. Ring junction proton H-4a was assumed as an *α*-orientation, and H-11a was *β*-orientation due to the coupling constant between these two protons. On the *β*-face, H-11a showed the NOESY cross peak with Me-18, in turn coupled with H-9 revealed an *α*-orientation of hydroxyl group at the C-9 position. Besides, NOESY correlations of H-19_A_/H-4a/H-8 unveiled *α*-orientation of H-8. Based on the above results, we could infer that Me-18 was a *β*-quasi-axial orientation, whereas H-7 and H_2_-19 were *α*-quasi-axial orientations. Thus, the relative stereochemistry of the cyclononane ring system was similar to that of asterolaurin A [4]. Additionally, the NOESY correlations of H-3 with H_2_-5 showed *β*-quasi-axial orientation revealed *α*-orientation of methoxyl group at C-3 position. Thus the structure of asterolaurin R was unambiguously established as shown in Figure 6.

The cytotoxicities of all isolated marine natural products (**1**–**10**) were evaluated in vitro against human breast (MCF-7), oral (Ca9-22), and ovarian (SK-OV-3) carcinomas. As illustrated in Table 2, compounds **1** (IC_50_ = 14.7 μM) and **2** (IC_50_ = 25.1 μM) selectively possessed strong activities against the MCF-7 cell. For the ovarian and oral cancer cells, all tested xenicane diterpenoids were inactive (>100 μM).

## 3. Experimental

### 3.1. General

Optical rotations were determined using a JASCO P-2100 polarimeter (Jasco, Tokyo, Japan), and IR spectra were recorded on a JASCO FT/IR-4600 infrared spectrometer (Jasco, Tokyo, Japan). NMR spectra were recorded on Varian 600 MHz NMR (Varian, Palo Alto, CA, USA) and Bruker AVIII-HD700X 700 MHz spectrometers (Bruker, Bremen, Germany). HRESIMS data were recorded on a VG Biotech Quattro 5022 mass spectrometer (VG Biotech, Altrincham, UK). GNPS data were obtained on an Agilent 6545XT AdvanceBio LC/Q-TOF mass spectrometer (Agilent Technologies, Santa Clara, CA, USA). Silica gel 60 (0.063–0.200 mm) was used for flash-column and open column chromatography (Merck KGaA, Darmstadt, Germany). Gel filtration column chromatography was performed with Sephadex LH-20 (GE Healthcare, Chicago, IL, USA). Precoated aluminum TLC plate/TLC silica gel 60 F_254_ were used for TLC analysis (Merck KGaA, Darmstadt, Germany). Normal-phase semi-preparative HPLC was accomplished using a Luna Silica (5 μm, 250 × 10 mm) column (Phenomenex, Torrance, CA, USA) on an L-6000 pump with an L-4000 UV detector (Hitachi, Tokyo, Japan), while reversed-phase HPLC was using Luna CN or Biphenyl (5 μm, 250 × 10 mm) columns (Phenomenex, Torrance, CA, USA) on a Chromaster 5110 pump with a Chromaster 5410 UV detector (Hitachi, Tokyo, Japan).

### 3.2. Animal Material

Specimens of soft coral *Asterospicularia laurae* were donated by Prof. Ya-Ching Shen in 2019. The animal materials were collected in August 2012 off the coast of Orchid Island, Taiwan. The samples were stored in a freezer until extraction. The material was identified by Prof. Dr. Jui-Hsin Su. A voucher sample (specimen code: AL001) was deposited at Department of Marine Biotechnology and Resources, National Sun Yat-sen University, Kaohsiung, Taiwan.

### 3.3. Global Natural Product Social Molecular Networking

Equal divisions (10 μL) of the MeOH-layer of Taiwanese soft coral *A. laurae* were dispensed into 96-well plates, dried under nitrogen, and resuspended in DMSO (10 μL), and 10 μL of a DMSO aliquot was injected into an Agilent 6545XT AdvanceBio LC/Q-TOF (quadrupole time-of-flight) equipped with an Agilent 1290 Infinity II LC system, eluting with an ACQUITY UPLC BEH C_18_ column (1.7 μm, 2.1 × 100 mm, flow rate: 0.4 mL/min, Waters). The elution program, using water (A) and acetonitrile (B), both with 0.1% formic acid as mobile phases, started with a 5% isocratic elution for 1 min and was then followed by a linear gradient from 5% to 99.5% B until 16 min, and then maintained 99.5% B as a solvent system for 10 min followed by re-equilibration period for 2 min before the next injection. The UPLC-Q-TOF-(-)MS/MS data acquired for all samples at a fixed collision energy of 40 eV were converted from RAW data files to mzXML file format using the ProteoWizard MSconvert software [23] and uploaded to the Global Natural Products Social Molecular Networking Web server to create a molecular network [24]. The resulting spectral networks were imported into Cytoscape version 3.8.2 [25]. Careful review of these GNPS data associated with the Comprehensive Marine Natural Products Database and Reaxys^®^ database highlighted a promising cluster (Figure 1 and Figure S45), as a possible source of new xenicane diterpenoids.

### 3.4. Extraction and Isolation

Soft coral *Asterospicularia laurae* (115.4 g, wet weight) was macerated with 95% ethanol (1.5 L, 3 times) at room temperature. The solvent was decanted and the extract was concentrated under reduced pressure to obtain a crude extract (31.4 g), which was partitioned between H_2_O and ethyl acetate to yield an ethyl acetate layer. The EtOAc layer was subsequently partitioned (hexanes/MeOH/H_2_O = 4:3:1) to obtain hexanes and MeOH layers. After ^1^H NMR, experiments were co-referred with TLC assay as well as GNPS MS/MS analysis on all obtained layers, and the MeOH layer was selected for further isolation. The MeOH layer (5.2 g) was chromatographed by a normal-phase silica gel flash column eluted with a gradient solvent system of hexanes and ethyl acetate (5:1~0:1) followed by stepwise ethyl acetate with methanol (20:1~0:1) to obtain six subfractions (LA1~6), according to TLC analysis. The third sub-fraction, LA3, was fractionated over Sephadex LH-20 using MeOH and CH_2_Cl_2_ (1:1) as a solvent to afford six subfractions (LA3-1~6). Fraction LA-3-5 was purified by normal-phase HPLC (hexane/dichloromethane/methanol, 50:45:5) to yield compounds **1** (2.7 mg), **3** (1.4 mg), **9** (1.6 mg), and **10** (2.3 mg). Fraction LA-3-4 was subjected to silica gel CC (CH_2_Cl_2_/MeOH, 1:0→0:1) to get five subfractions (LA3-4-1~5), and subfraction LA3-4-4 was further separated by normal-phase HPLC (hexane/dichloromethane/methanol, 57:38:5) to afford compounds **4** (1.4 mg), **7** (1.3 mg), and **8** (2.1 mg). Besides, Fr. LA3-4-3 was isolated by reverse-phase HPLC using a CN column and gave compound **2** (1.7 mg) along with two subfractions Fr. LA-3-4-3-1~2. The fraction LA-3-4-3-1 was isolated by RP-Biphenyl HPLC (methanol/H_2_O, 60/40) to give compounds **5** (0.5 mg) and **6** (0.5 mg).

### 3.5. Spectroscopic Data

Asterolaurin O (**1**) amorphous, colorless gum, ${\left[\alpha \right]}_{D}^{26}$ −1.0° (*c* 0.05, MeOH); IR (neat) ν_max_ 3424, 2960, 2929, 1719, 1379, 1261, 1167, 1033 cm^−1^; ^1^H-NMR and ^13^C-NMR (CD_3_OD, 700/175 MHz) see Table 1; HRESIMS *m/z* 451.10903 (calcd for C_20_H_29_BrNaO_5_, 451.10906).
Asterolaurin P (**2**) pale yellowish amorphous gum, ${\left[\alpha \right]}_{D}^{26}\;$−47.6° (*c* 0.05, MeOH); IR (neat) *ν*_max_ 3454, 2926, 1727, 1455, 1263, 1029 cm^−1^; ^1^H-NMR and ^13^C-NMR (CD_3_OD, 600/150 MHz) see Table 1; HRESIMS *m/z* 369.20379 (calcd for C_21_H_30_O_4_Na, 369.20363).
Asterolaurin Q (**3**) amorphous, colorless gum, ${\left[\alpha \right]}_{D}^{26}$ −1.0° (*c* 0.05, MeOH); IR (neat) ν_max_ 3420, 2962, 2927, 2360, 1703, 1638, 1261, 1091 cm^−1^; ^1^H-NMR and ^13^C-NMR (CD_3_OD, 700/175 MHz) see Table 1; HRESIMS *m/z* 371.18294 (calcd for C_20_H_28_O_5_Na, 371.18290).
Asterolaurin R (**4**) amorphous, colorless gum, ${\left[\alpha \right]}_{D}^{26}$ −47.6° (*c* 0.05, MeOH); IR (neat) *ν*_max_ 3433, 2964, 1643, 1260, 1072 cm^−1^; ^1^H-NMR and ^13^C-NMR (CDCl_3_, 600/150 MHz) see Table 1; HRESIMS *m/z* 387.21425 (calcd for C_21_H_32_O_5_Na, 387.21420).

### 3.6. Cytotoxic Assays

Breast (MCF-7), oral (Ca9-22), and ovarian (SK-OV-3) cancer cell lines were available from the American Type Culture Collection (ATCC, Manassas, VA, USA) or the Japanese Collection of Research Bioresources (JCRB) Cell Bank (National Institute of Biomedical Innovation, Osaka, Japan). The cell viability was detected by MTS assay at 72 h treatment as previously described [24].

## 4. Conclusions

With the assistance of molecular networking-based de-replication strategy, xenicane diterpenes were targeted and obtained from the marine soft coral *A. laurae*. Among ten isolated compounds, asterolaurins O–Q (**1**–**3**) were identified as new xeniolides (possessing a δ-lactone-cyclononane skeleton), and asterolaurin R (**4**) was a new xenicin (containing an 11-oxabicyclo[7.4.0]tridecane ring system with an acetal functionality). It is noteworthy that asterolaurin O (**1**) was the first case of natural brominated tricarbocyclic floridicins yielded from the family Xeniidae. Moreover, compared with other asterolaurins obtained from the genus *Asterospicularia*, asterolaurin O (**1**) showed potent inhibition toward MCF-7 cells. This finding suggests that brominated xenicane-type diterpenes were worthy for further cytotoxic evaluations.

## Figures and Tables

**Table 1:** ^1^H-NMR and ^13^C-NMR data for compounds **1**–**4.**

	1 ^b^		2 ^a^		3 ^b^		4 ^c^	
	δ_H_ (*J* in Hz)	δc	δ_H_ (*J* in Hz)	δc	δ_H_ (*J* in Hz)	δc	δ_H_ (*J* in Hz)	δc
1		176.9, s	4.11, dd (5.9, 11.3)	72.4, t	4.09, dd (4.1, 11.0)	71.5, t	3.61, dd (6.5, 11.5)	65.9, t
			3.63, t (11.3)		3.91, t (11.0)		3.28, t (11.5)	
3	4.44, d (12.0)	73.2, t		171.4, s		172.2, s	5.17, brs	99.2, d
	5.06, d (12.0)							
4		137.0, s		134.6, s		133.3, s		138.5, s
4a	3.18, t (12.0)	39.3, d	2.70, dt (2.9, 11.0)	52.0, d	3.17, t (9.2)	42.8, d	2.97, brd (11.5)	43.1, d
5	1.95, m	33.4, t	1.61, m	39.1, t	1.84, m	38.5, t	1.61, m	34.8, t
	1.87, m						1.83, m	
6*α*	2.08, m	40.8, t	2.19, m	40.9, t	1.92, m	31.0, t	2.20, t (3.6)	40.5, t
6*β*	1.82, m						2.22, t (3.6)	
7		37.4, s		133.2, s		149.0, s		59.2, s
8	4.09, d (5.7)	71.2, d	5.26, d (7.4)	131.8, d	3.97, d (9.0)	83.1, d	3.00, d (8.0)	66.9, d
9	4.37, td (5.7, 8.7)	75.1, d	4.72, t (7.4)	67.9, d	4.06, dd (3.9, 9.0)	70.5, d	3.80, dd (8.0, 7.4)	69.1, d
10*α*	2.24, d (8.7)	39.8, t	2.34, d (13.6)	46.2, t	2.48, m	45.0, t	2.45, m	44.7, t
10*β*	2.22, d (5.7)		2.50, dd (13.6, 6.4)		2.60, m		2.47, t (7.4)	
11		72.5, s		149.5, s		148.3, s		147.4, s
11a	2.88, d (12.0)	57.3, d	2.06, dd (5.9, 11.0)	51.1, d	2.46, m	44.7, d	2.26, dd (6.5, 11.5)	50.7, d
12	6.15, d (11.1)	129.7, d	6.53, d (11.0)	137.3, d	7.04, d (11.8)	140.4, d	6.36, d (11.3)	126.5, d
13	6.34, dd (11.1, 15.3)	122.3, d	6.76, dd (11.0, 15.7)	126.6, d	6.57, dd (11.8, 15.1)	121.7, d	6.44, dd (11.3,14.9)	120.4, d
14	5.94, d (15.3)	146.2, d	5.98, d (15.7)	146.4, d	6.35, d (15.1)	153.2, d	5.97, d (14.9)	144.6, d
15		71.3, s		76.5, s		71.5, s		71.0, s
16	1.30, s	29.8, q	1.30, s	26.0, q	1.34, s	29.7, q	1.32, s	29.9, q
17	1.30, s	29.8, q	1.30, s	25.9, q	1.34, s	29.7, q	1.32, s	29.9, q
18	1.15, s	34.7, q	1.70, s	17.3, q	5.20, d (1.6)	120.2, t	1.44, s	17.3, q
					5.10, d (1.6)			
19_A_	1.76, d (14.6)	44.7, t	5.06, s	115.3, t	5.02, d (1.8)	116.5, t	4.85, s	114.8, t
19_B_	1.84, d (14.6)		4.95, s		4.84, d (1.8)		5.11, s	
OH	4.61, brs							
OMe			3.16, s	50.9, q			3.47, s	55.1, q

^a 1^H and ^13^C-NMR were measured in MeOH-*d*4 at 600 and 150 MHz, respectively. ^b 1^H and ^13^C-NMR were measured in MeOH-*d*4 at 700 and 175 MHz, respectively. ^c 1^H and ^13^C-NMR were measured in CDCl_3_ at 600 and 150 MHz, respectively.

**Table 2:** Results of Cytotoxicities (IC_50_, µM) of isolated compounds **1**–**10**.

Compound/Tumor Cells	MCF-7	Ca9-22	SK-OV-3
**1**	14.7 ± 0.23	>100	>100
**2**	25.1 ± 4.1	>100	>100
**3**	>100	>100	>100
**4**	>100	>100	>100
**5**	>100	>100	>100
**6**	>100	>100	>100
**7**	>100	>100	>100
**8**	>100	>100	>100
**9**	>100	>100	>100
**10**	>100	>100	>100
**Cisplatin ^a^**	19.8		13.8

^a^ Positive control; data come from literatures [21,22].

## Data Availability

Data are contained within the article and Supplementary Material.

## Supplementary Materials

The HRESIMS, IR spectra, ^1^H, ^13^C, DEPT, HSQC, COSY, HMBC, and NOESY spectra of compounds **1**–**4** are available online at https://www.mdpi.com/1660-3397/19/3/123/s1, Figure S1. The HRESIMS of asterolaurin O (**1**); Figure S2. The IR spectrum of asterolaurin O (**1**); Figure S3. The ^1^H-NMR spectrum of asterolaurin O (**1**) (700 MHz in CD_3_OD); Figure S4. The ^1^H-NMR spectrum (0.5-2.5 ppm) of asterolaurin O; Figure S5. The ^13^C-NMR spectrum of asterolaurin O (**1**) (175 MHz in CD_3_OD); Figure S6. The ^13^C-NMR spectrum (20-55 ppm) of asterolaurin O (**1**); Figure S7. The DEPT spectrum of asterolaurin O (**1**); Figure S8. The ^1^H-^1^H COSY spectrum of asterolaurin O (**1**); Figure S9. The HSQC spectrum of asterolaurin O (**1**); Figure S10. The HMBC spectrum of asterolaurin O (**1**); Figure S11. The NOESY spectrum of asterolaurin O (**1**); Figure S12. The HRESIMS of asterolaurin P (**2**); Figure S13. The IR spectrum of asterolaurin P (**2**); Figure S14. The ^1^H-NMR spectrum of asterolaurin P (**2**) (600 MHz in CD_3_OD); Figure S15. The ^1^H-NMR spectrum (0.5-2.5 ppm) of asterolaurin P; Figure S16. The ^13^C-NMR spectrum of asterolaurin P (**2**) (150 MHz in CD_3_OD); Figure S17. The ^13^C-NMR spectrum (15-55 ppm) of asterolaurin P (**2**); Figure S18. The DEPT spectra of asterolaurin P (**2**); Figure S19. The ^1^H-^1^H COSY spectrum of asterolaurin P (**2**); Figure S20. The HSQC spectrum of asterolaurin P (**2**); Figure S21. The HMBC spectrum of asterolaurin P (**2**); Figure S22. The NOESY spectrum of asterolaurin P (**2**); Figure S23. The HRESIMS of asterolaurin Q (**3**); Figure S24. The IR spectrum of asterolaurin Q (**3**); Figure S25. The ^1^H-NMR spectrum of asterolaurin Q (**3**) (700 MHz in CD_3_OD); Figure S26. The ^1^H-NMR spectrum (0.5-2.5 ppm) of asterolaurin Q; Figure S27. The ^13^C-NMR spectrum of asterolaurin Q (**3**) (175 MHz in CD_3_OD); Figure S28. The ^13^C-NMR spectrum (20-55 ppm) of asterolaurin Q; Figure S29. The DEPT spectrum of asterolaurin Q (**3**); Figure S30. The ^1^H-^1^H COSY spectrum of asterolaurin Q (**3**); Figure S31. The HSQC spectrum of asterolaurin Q (**3**); Figure S32. The HMBC spectrum of asterolaurin Q (**3**); Figure S33. The NOESY spectrum of asterolaurin Q (**3**); Figure S34. The HRESIMS of asterolaurin R (**4**); Figure S35. The IR spectrum of asterolaurin R (**4**); Figure S36. The ^1^H-NMR spectrum of asterolaurin R (**4**) (600 MHz in CDCl_3_); Figure S37. The ^1^H-NMR spectrum (0.5-2.5 ppm) of asterolaurin R; Figure S38. The ^13^C-NMR spectrum of asterolaurin R (**4**) (150 MHz in CDCl_3_); Figure S39. The ^13^C-NMR spectrum (15-55 ppm) of asterolaurin R; Figure S40. The DEPT spectra of asterolaurin R; Figure S41. The ^1^H-^1^H COSY spectrum of asterolaurin R (**4**); Figure S42. The HSQC spectrum of asterolaurin R (**4**); Figure S43. The HMBC spectrum of asterolaurin R (**4**); Figure S44. The NOESY spectrum of asterolaurin R (**4**); Figure S45. Negative Q-TOF MS/MS spectrum of xeniolide-A (**5**).
