# Supplementary material for: Targeted Isolation of Xenicane Diterpenoids From Taiwanese Soft Coral Asterospicularia laurae
Source: Mar Drugs. 2021 Feb 25;19(3):123. doi: 10.3390/md19030123 (PMC7996537; doi:10.3390/md19030123)
Supplement: Supplementary file 1 [file marinedrugs-19-00123-s001.pdf]

SUPPLEMENTARY MATERIAL

New xenicane diterpenoid from Taiwanese soft coral *Asterospicularia laurae*

Yu-Chi Lin [1], Yi-Jen Chen [2], Shu-Rong Chen [3], Wan-Ju Lien [4], Hsueh-Wei Chang [4,5,6,7], Yu-Liang Yang [8], Chia-Ching Liaw [9,10], Jui-Hsin Su [11], Ching-Yeu Chen [12,*], and Yuan-Bin Cheng [1,2,3,*]

1 Department of Marine Biotechnology and resources, National Sun Yat-sen University, Kaohsiung 804351, Taiwan; m8952612@hotmail.com

2 Department of Fragrance and Cosmetic Science, College of Pharmacy, Kaohsiung Medical University, Kaohsiung 807378, Taiwan; mermaid190111@gmail.com

3 Graduate Institute of Natural Products, Center for Natural Product Research and Development, College of Pharmacy, Kaohsiung Medical University, Kaohsiung 807378, Taiwan; highshorter@hotmail.com

4 Department of Biomedical Science and Environmental Biology, PhD program in Life Science, College of Life Science, Kaohsiung Medical University, Kaohsiung 80708, Taiwan; rfsl.lien@gmail.com (W.-J.L.); changhw@kmu.edu.tw (H.-W.C.)

5 Center for Cancer Research, Kaohsiung Medical University, Kaohsiung 80708, Taiwan

6 Cancer Center, Kaohsiung Medical University Hospital, Kaohsiung 80708, Taiwan

7 Department of Medical Research, Kaohsiung Medical University Hospital, Kaohsiung, Taiwan

8 Agricultural Biotechnology Research Center, Academia Sinica, Taipei 115, Taiwan; ylyang@gate.sinica.edu.tw

9 Division of Chinese Materia Medica Development, National Research Institute of Chinese Medicine, Taipei 11221, Taiwan; liawcc@nricm.edu.tw

10 Department of Biochemical Science and Technology, National Chiayi University, Chiayi 60004, Taiwan

11 Graduate Institute of Marine Biology, National Dong Hwa University, Pingtung 944401, Taiwan; x2219@nmmba.gov.tw

12 Department of Physical Therapy, Tzu-Hui Institute of Technology, Pingtung 92641, Taiwan

* Correspondence: chingyeu1971@yahoo.com.tw (C.-Y.C.); jmb@mail.nsysu.edu.tw (Y.-B.C.); Tel.: +886-8-779-9821-8639 (C.-Y.C.); +886-07-525-2000 ext. 5212 (Y.-B.C.)

Contents

Figure S1. The HRESIMS of asterolaurin O (**1**)

Figure S2. The IR spectrum of asterolaurin O (**1**)

Figure S3. The ^{1}H-NMR spectrum of asterolaurin O (**1**) (700 MHz in CD_3OD)

Figure S4. The ^{1}H-NMR spectrum (0.5-2.5 ppm) of asterolaurin O

Figure S5. The ^{13}C-NMR spectrum of asterolaurin O (**1**) (175 MHz in CD_3OD)

Figure S6. The ^{13}C-NMR spectrum (20-55 ppm) of asterolaurin O (**1**)

Figure S7. The DEPT spectrum of asterolaurin O (**1**)

Figure S8. The ^{1}H-^{1}H COSY spectrum of asterolaurin O (**1**)

Figure S9. The HSQC spectrum of asterolaurin O (**1**)

Figure S10. The HMBC spectrum of asterolaurin O (**1**)

Figure S11. The NOESY spectrum of asterolaurin O (**1**)

Figure S12. The HRESIMS of asterolaurin P (**2**)

Figure S13. The IR spectrum of asterolaurin P (**2**)

Figure S14. The ^{1}H-NMR spectrum of asterolaurin P (**2**) (600 MHz in CD_3OD)

Figure S15. The ^{1}H-NMR spectrum (0.5-2.5 ppm) of asterolaurin P

Figure S16. The ^{13}C-NMR spectrum of asterolaurin P (**2**) (150 MHz in CD_3OD)

Figure S17. The ^{13}C-NMR spectrum (15-55 ppm) of asterolaurin P (**2**)

Figure S18. The DEPT spectra of asterolaurin P (**2**)

Figure S19. The ^{1}H-^{1}H COSY spectrum of asterolaurin P (**2**)

Figure S20. The HSQC spectrum of asterolaurin P (**2**)

Figure S21. The HMBC spectrum of asterolaurin P (**2**)

Figure S22. The NOESY spectrum of asterolaurin P (**2**)

Figure S23. The HRESIMS of asterolaurin Q (**3**)

Figure S24. The IR spectrum of asterolaurin Q (**3**)

Figure S25. The ^{1}H-NMR spectrum of asterolaurin Q (**3**) (700 MHz in CD_3OD)

Figure S26. The ^{1}H-NMR spectrum (0.5-2.5 ppm) of asterolaurin Q

Figure S27. The ^{13}C-NMR spectrum of asterolaurin Q (**3**) (175 MHz in CD_3OD)

Figure S28. The ^{13}C-NMR spectrum (20-55 ppm) of asterolaurin Q

Figure S29. The DEPT spectrum of asterolaurin Q (**3**)

Figure S30. The ^{1}H-^{1}H COSY spectrum of asterolaurin Q (**3**)

Figure S31. The HSQC spectrum of asterolaurin Q (**3**)

Figure S32. The HMBC spectrum of asterolaurin Q (**3**)

Figure S33. The NOESY spectrum of asterolaurin Q (**3**)

Figure S34. The HRESIMS of asterolaurin R (**4**)

Figure S35. The IR spectrum of asterolaurin R (**4**)

Figure S36. The ^{1}H-NMR spectrum of asterolaurin R (**4**) (600 MHz in $CDCl_3$)

Figure S37. The ^{1}H-NMR spectrum (0.5-2.5 ppm) of asterolaurin R

Figure S38. The ^{13}C-NMR spectrum of asterolaurin R (**4**) (150 MHz in $CDCl_3$)

Figure S39. The ^{13}C-NMR spectrum (15-55 ppm) of asterolaurin R

Figure S40. The DEPT spectra of asterolaurin R

Figure S41. The ^{1}H-^{1}H COSY spectrum of asterolaurin R (**4**)

Figure S42. The HSQC spectrum of asterolaurin R (**4**)

Figure S43. The HMBC spectrum of asterolaurin R (**4**)

Figure S44. The NOESY spectrum of asterolaurin R (**4**)

Figure S45. Negative Q-TOF MS/MS spectrum of xeniolide-A (**5**)

Mass Spectrum SmartFormula Report

Analysis Info

Analysis Name	D:\Data\g4\HS-35-H2_000012.d	6/11/2020 3:51:49 PM
Method	broadband first signal	Operator: YU HSIAO-CHING
Sample Name	HS-35-H2	Instrument: BRUKER FT-MS solariX
Comment	ESI Positive	

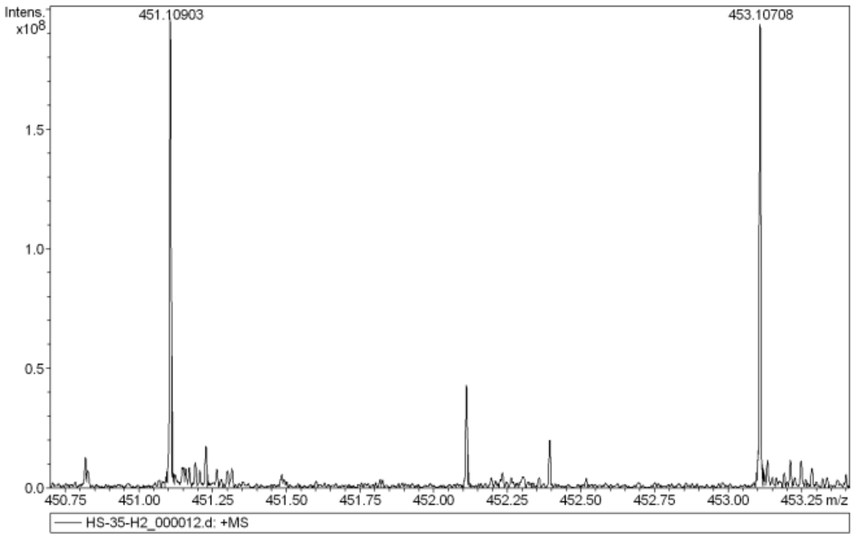

Meas. m/z	#	Formula	Score	m/z	err [mDa]	err [ppm]	mSigma	rdb	e⁻ Conf	N-Rule
451.10903	1	C 20 H 29 Br Na O 5	100.00	451.10906	0.02	0.05	8.4	5.5	even	ok

Figure S1. The HRESIMS of asterolaurin O (**1**)

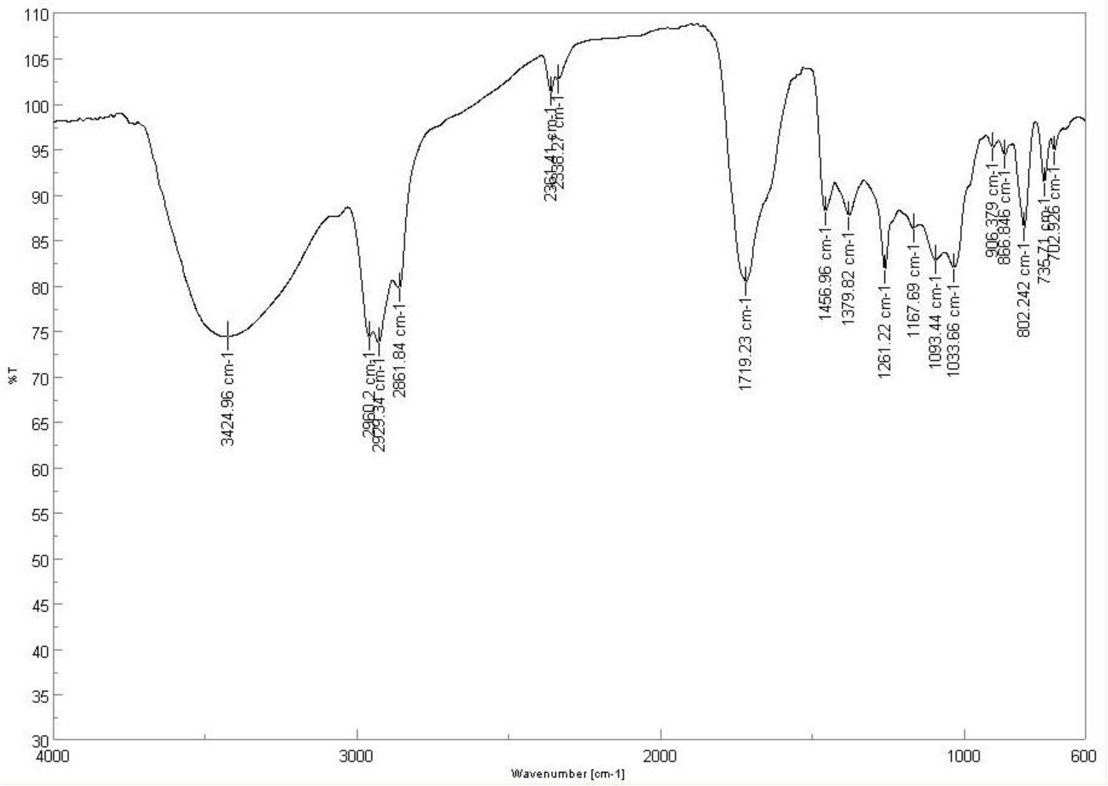

Figure S2. The IR spectrum of asterolaurin O (**1**)

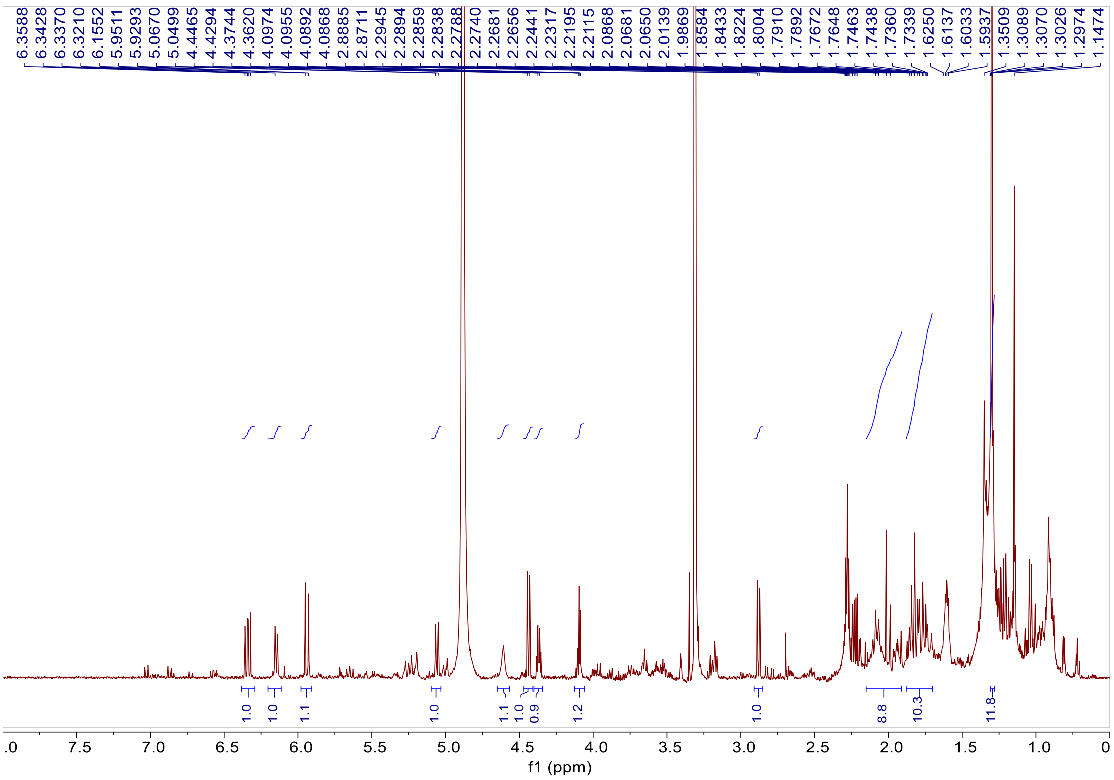

Figure S3. The ^{1}H-NMR spectrum of asterolaurin O (**1**) (700 MHz in CD_3OD)

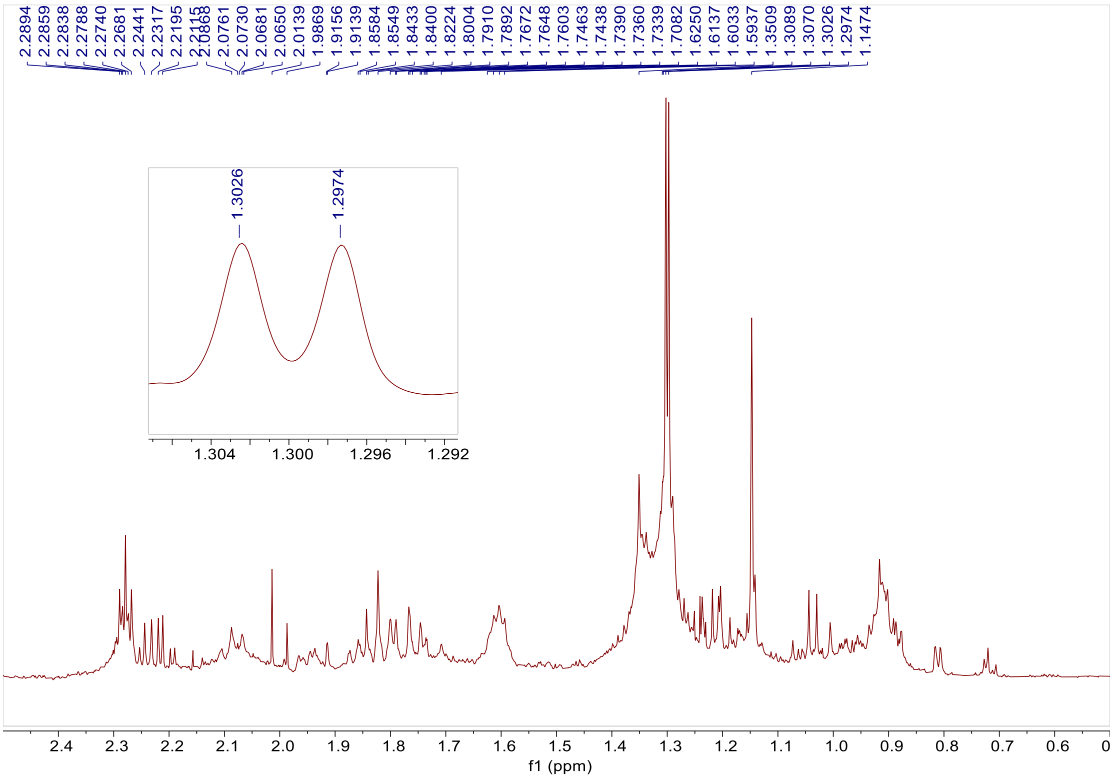

Figure S4. The ^{1}H-NMR spectrum (0.5-2.5 ppm) of asterolaurin O

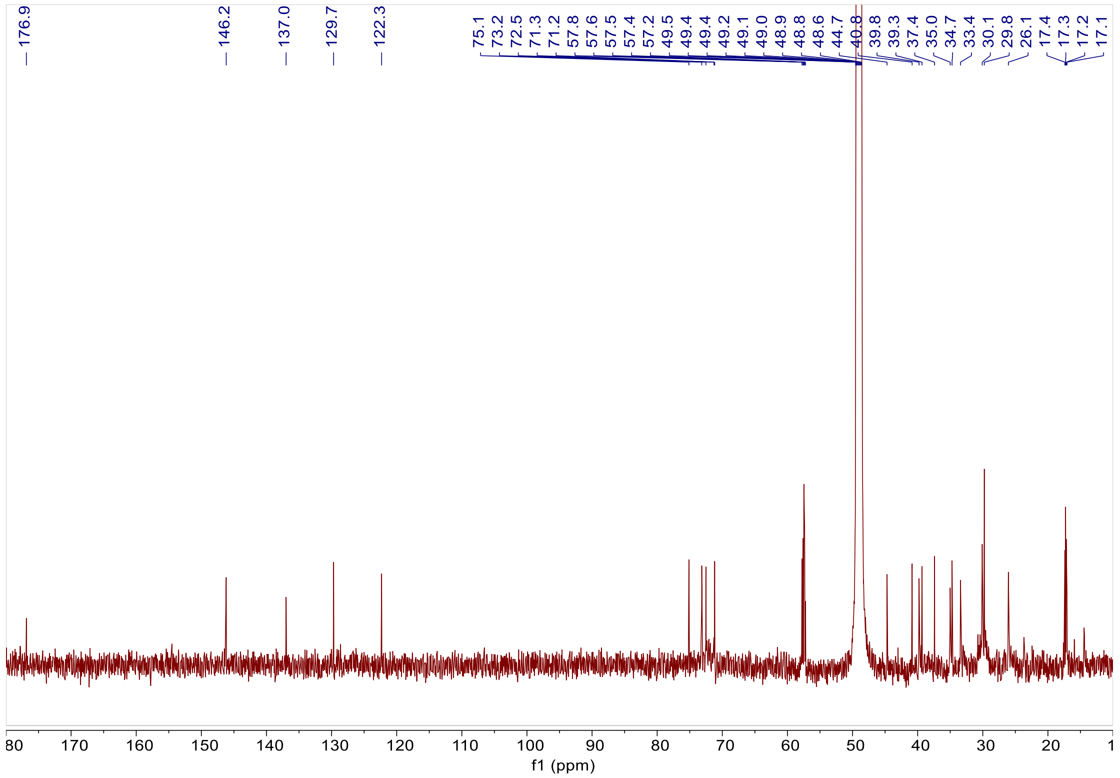

Figure S5. The ^{13}C-NMR spectrum of asterolaurin O (**1**) (175 MHz in CD_3OD)

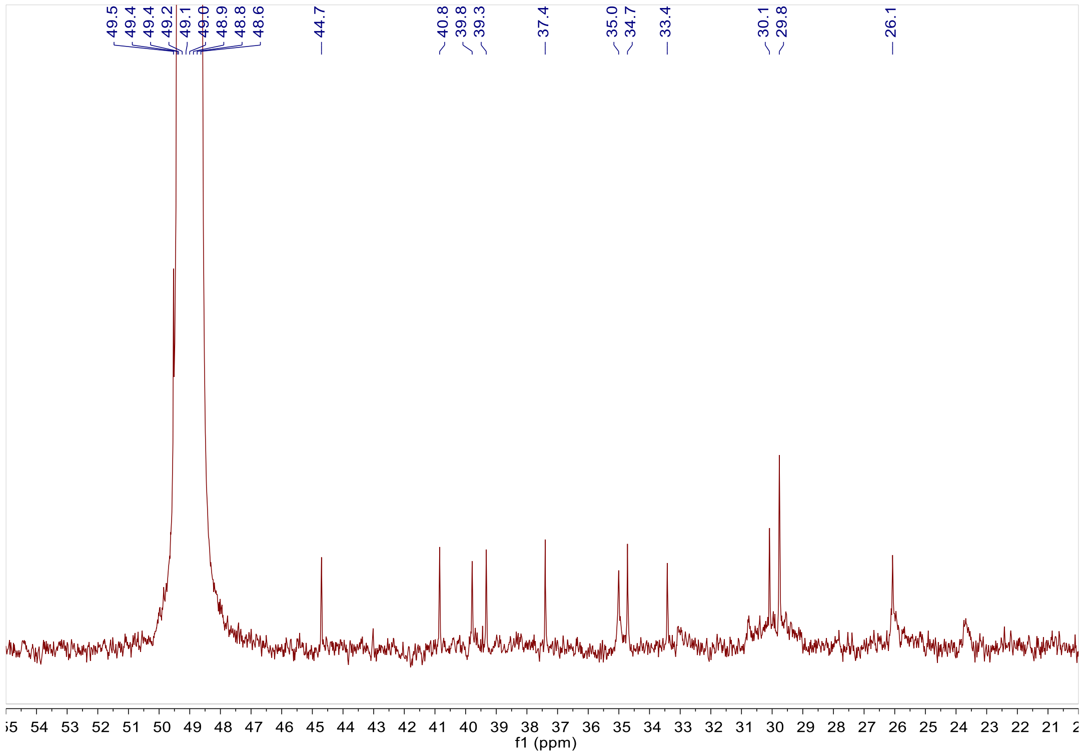

Figure S6. The ^{13}C-NMR spectrum (20-55 ppm) of asterolaurin O (**1**)

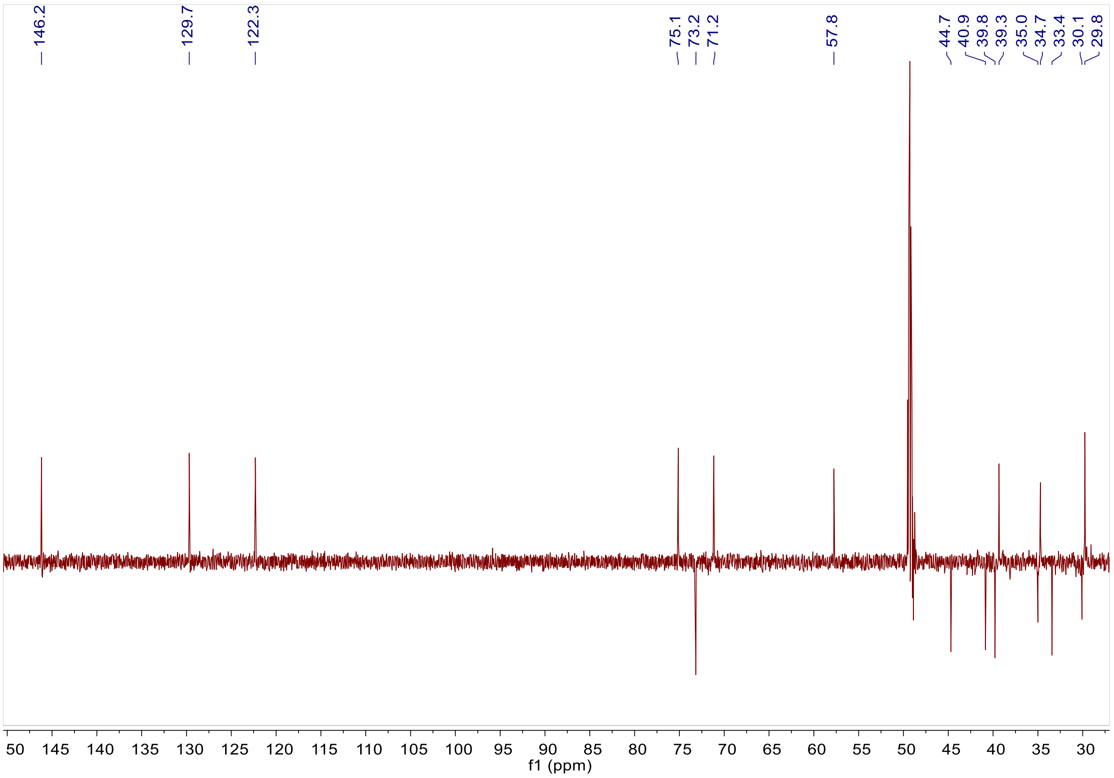

Figure S7. The DEPT spectrum of asterolaurin O (**1**)

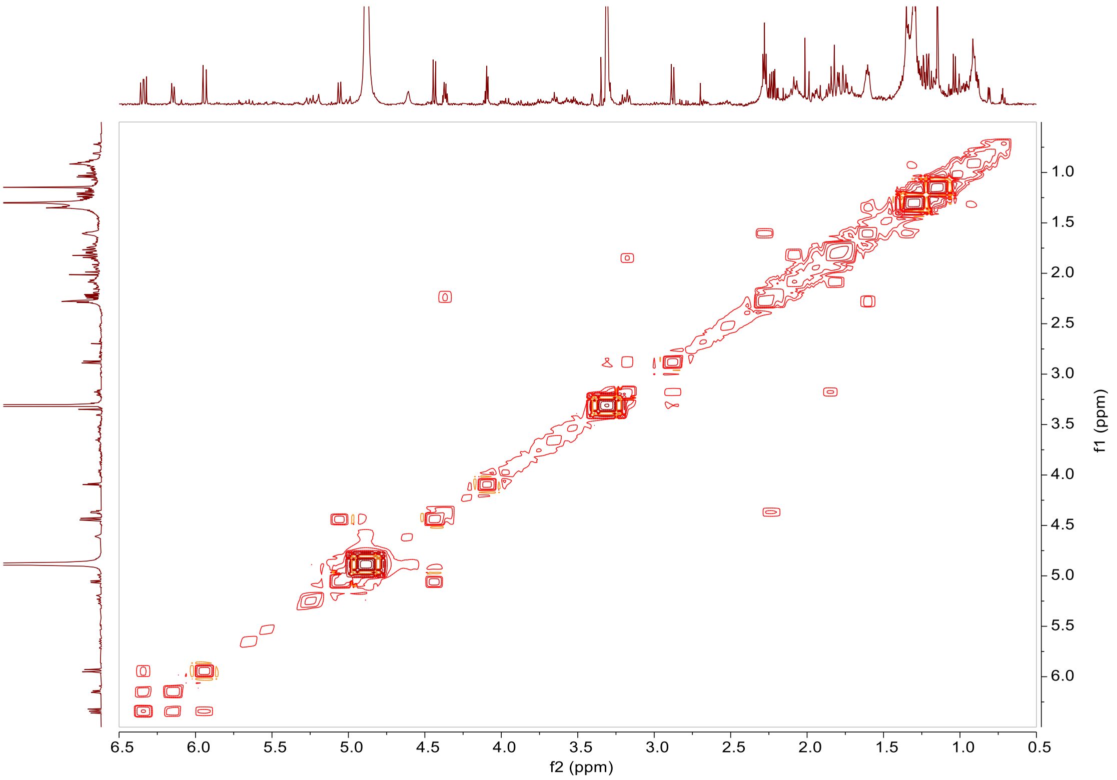

Figure S8. The ^{1}H-^{1}H COSY spectrum of asterolaurin O (**1**)

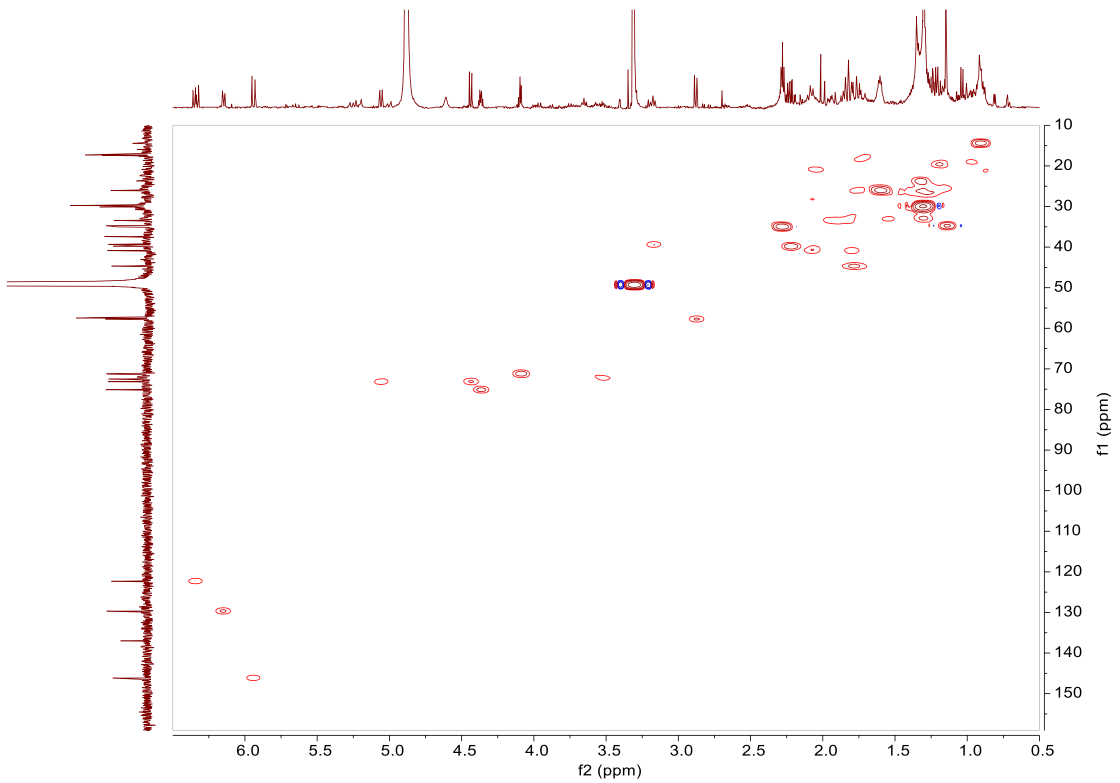

Figure S9. The HSQC spectrum of asterolaurin O (**1**)

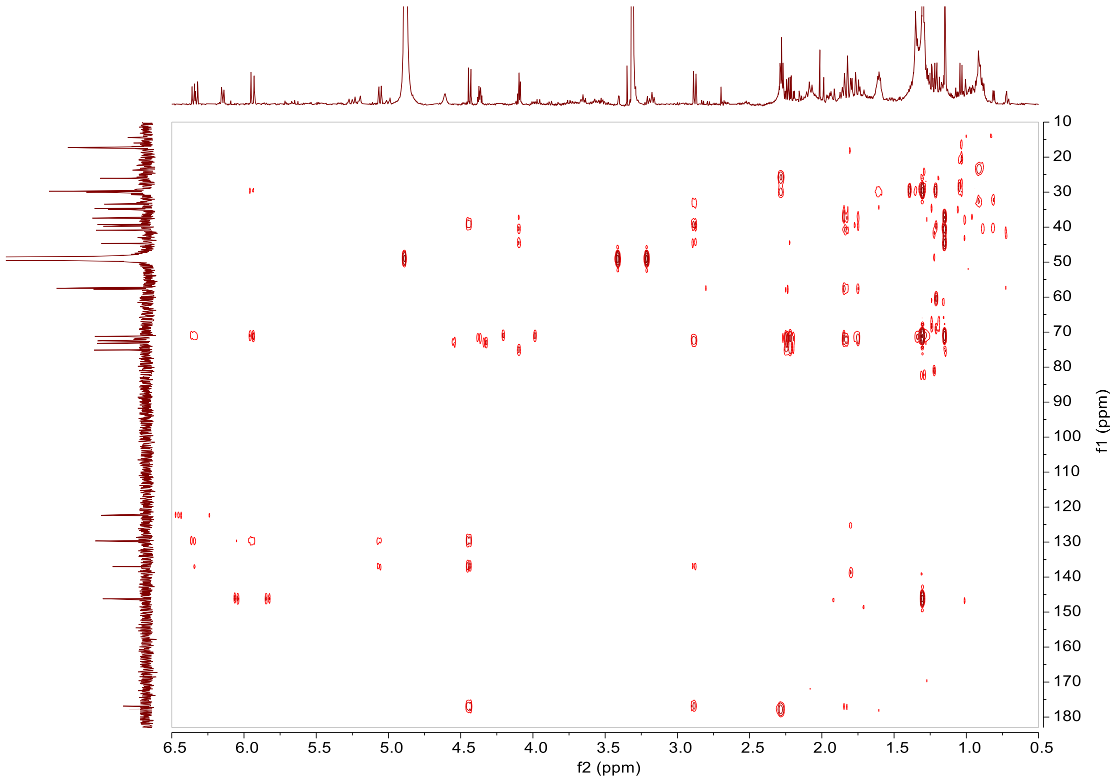

Figure S10. The HMBC spectrum of asterolaurin O (**1**)

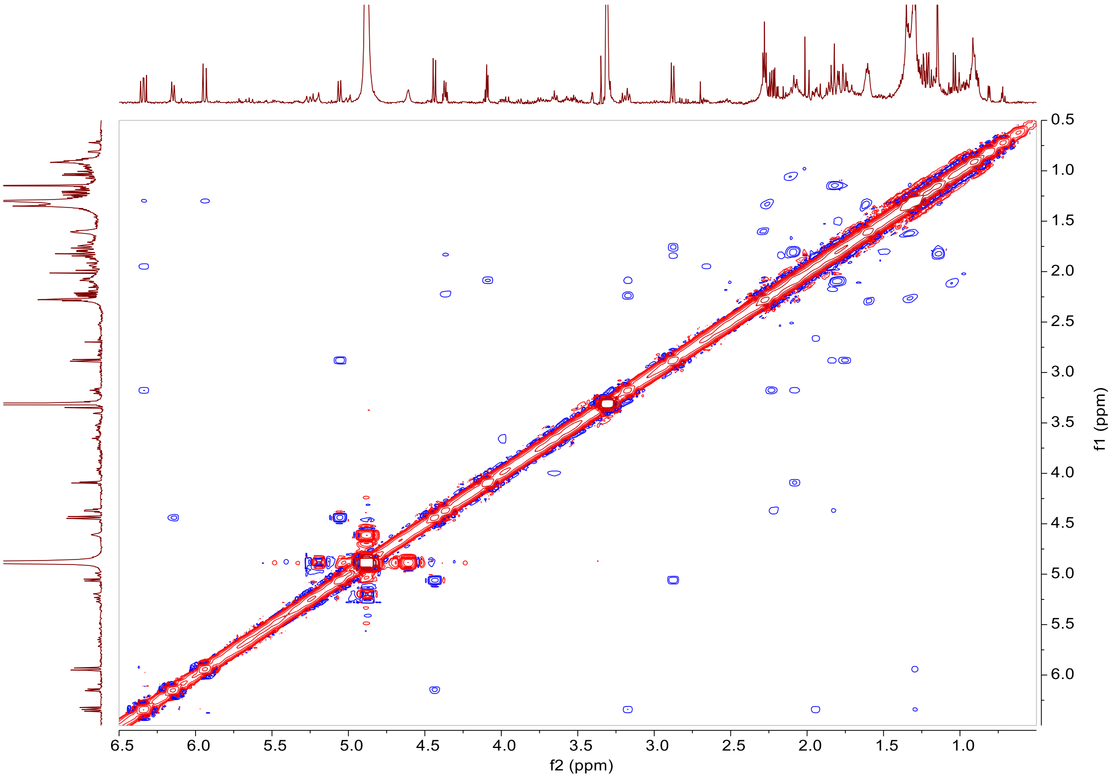

Figure S11. The NOESY spectrum of asterolaurin O (**1**)

Mass Spectrum SmartFormula Report

Analysis Info

Analysis Name	D:\Data\g4\HSA_000003.d	3/20/2020 3:43:10 PM
Method	broadband first signal	Operator: YU HSIAO-CHING
Sample Name	HS-A	Instrument: BRUKER FT-MS solariX
Comment	ESI Positive	

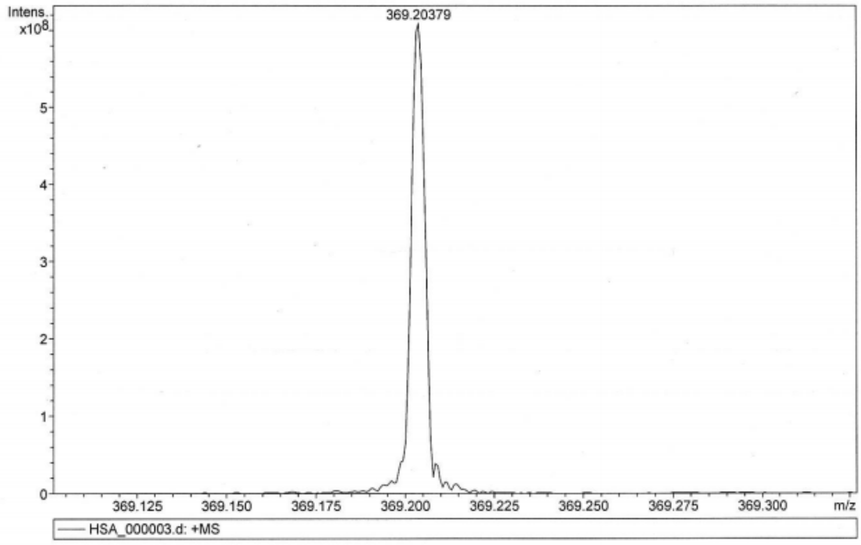

Meas. m/z	#	Formula	Score	m/z	err [mDa]	err [ppm]	mSigma	rdb	e⁻ Conf	N-Rule
369.20379	1	C 21 H 30 Na O 4	100.00	369.20363	-0.16	-0.43	8.4	6.5	even	ok

Figure S12. The HRESIMS of asterolaurin P (**2**)

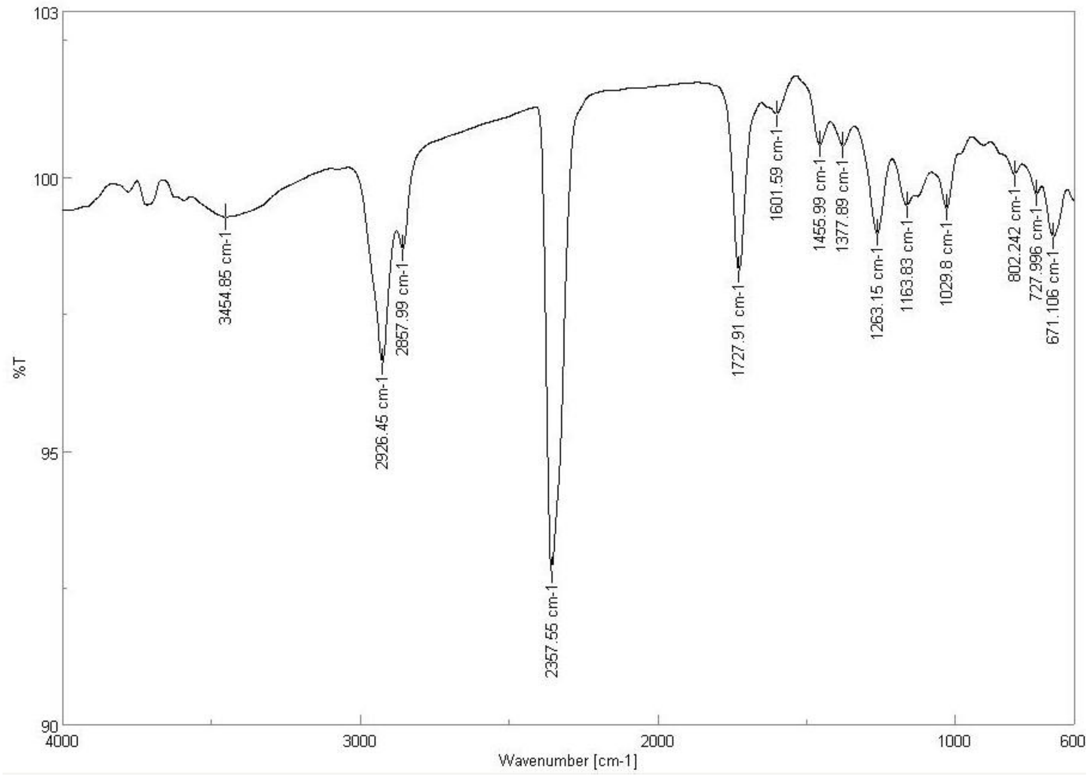

Figure S13. The IR spectrum of asterolaurin P (**2**)

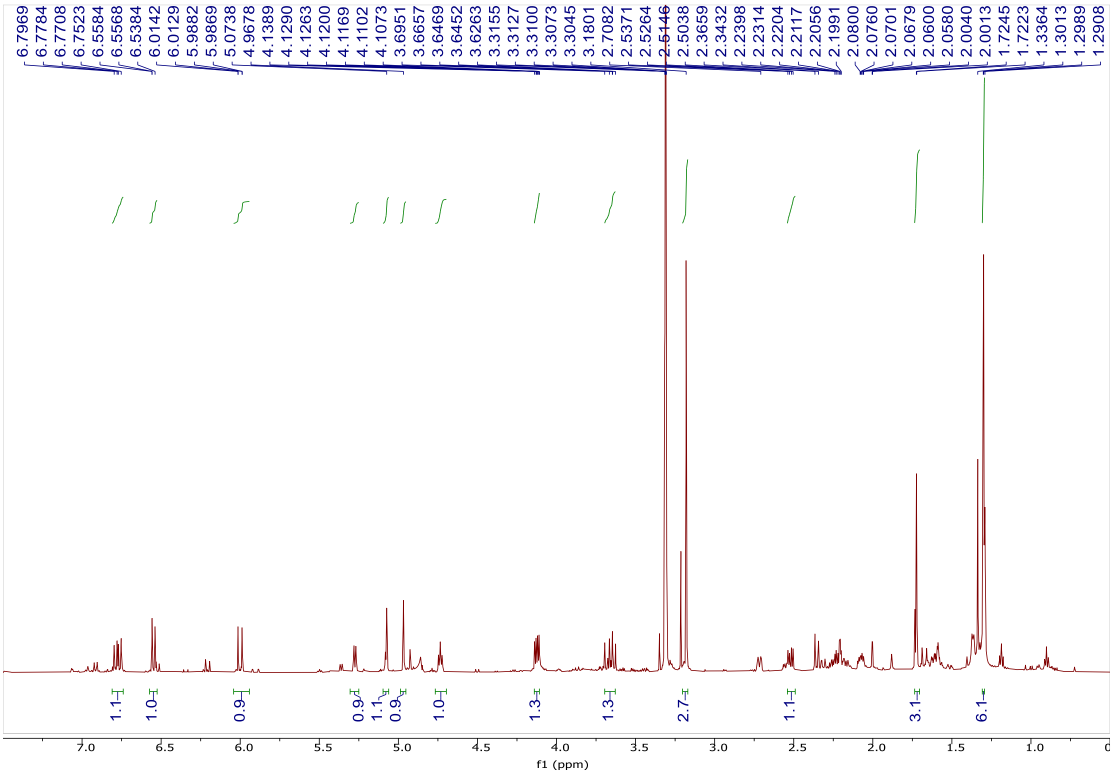

Figure S14. The ^{1}H-NMR spectrum of asterolaurin P (**2**) (600 MHz in CD_3OD)

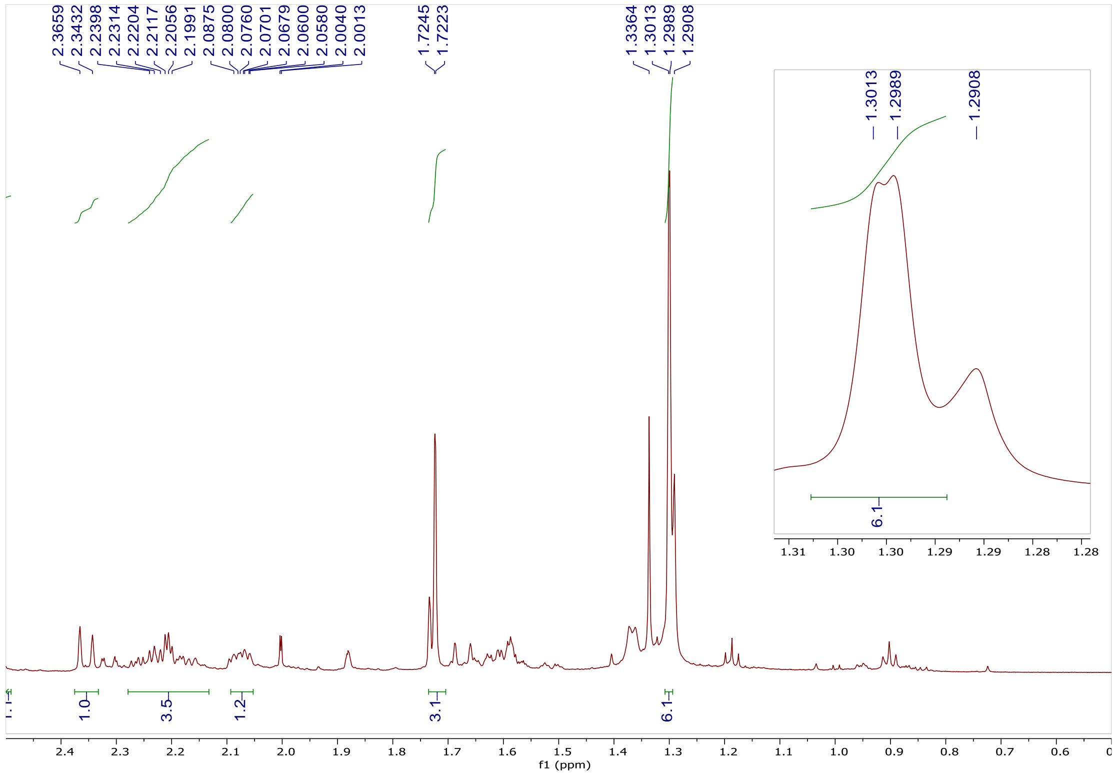

Figure S15. The ^{1}H-NMR spectrum (0.5-2.5 ppm) of asterolaurin P

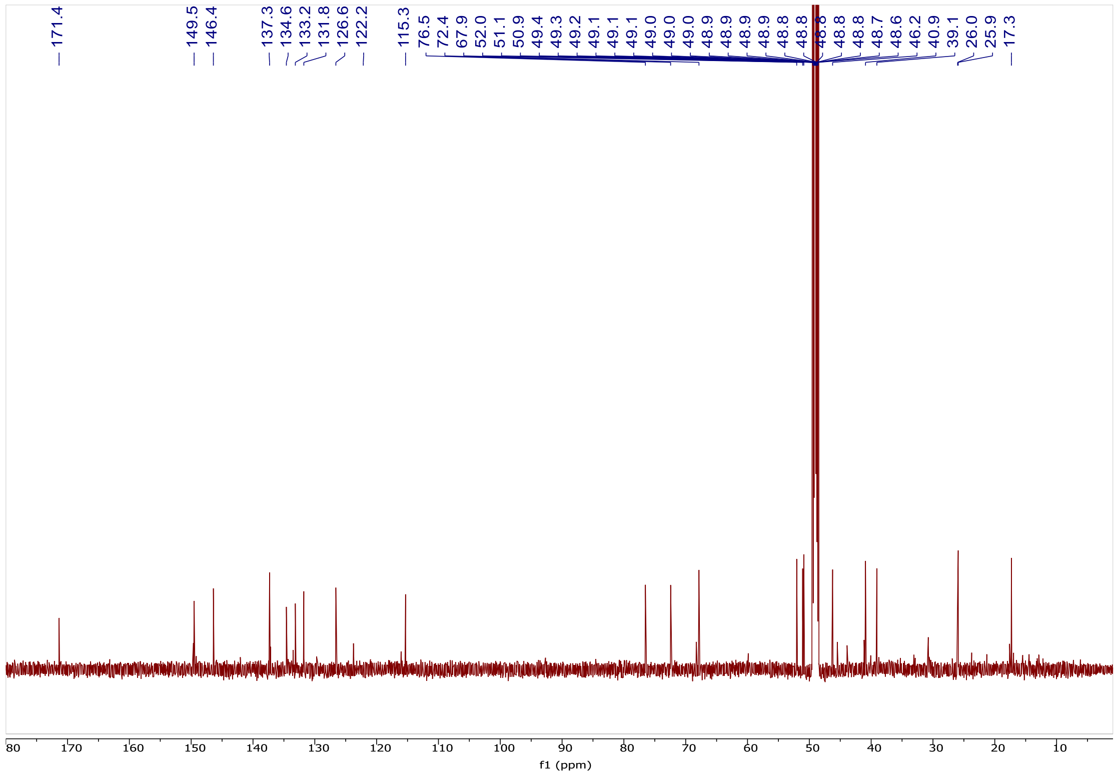

Figure S16. The ^{13}C-NMR spectrum of asterolaurin P (**2**) (150 MHz in CD_3OD)

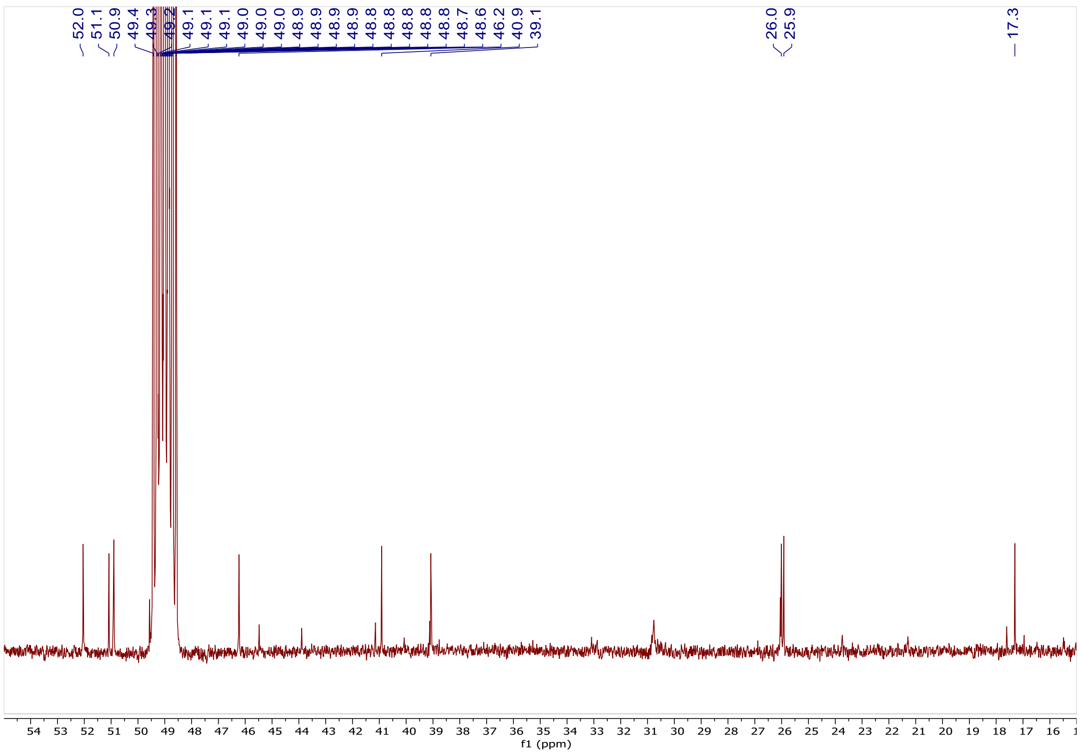

Figure S17. The ^{13}C-NMR spectrum (15-55 ppm) of asterolaurin P (**2**)

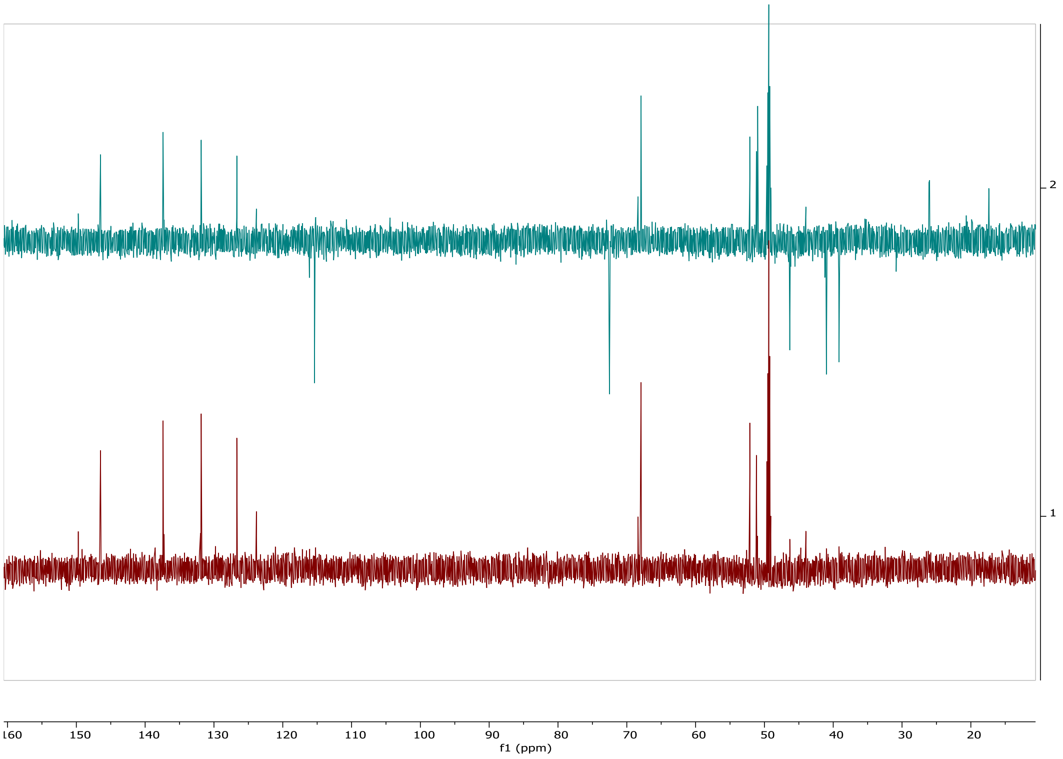

Figure S18. The DEPT spectra of asterolaurin P (**2**)

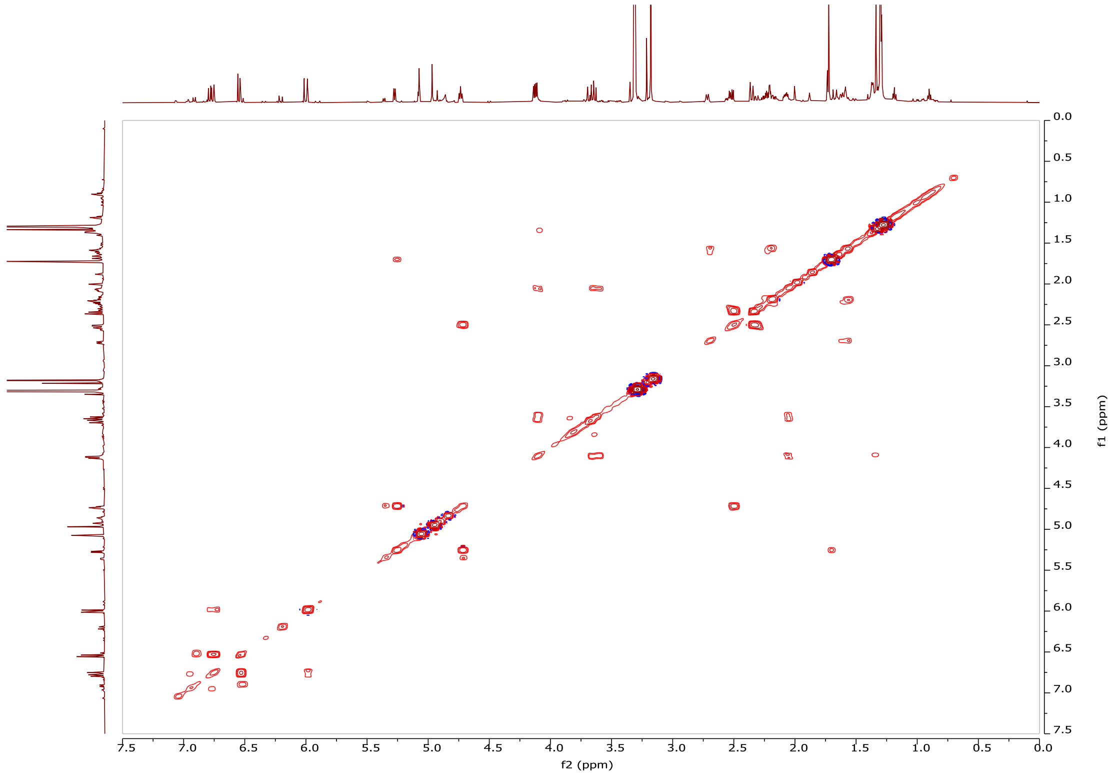

Figure S19. The ^{1}H-^{1}H COSY spectrum of asterolaurin P (**2**)

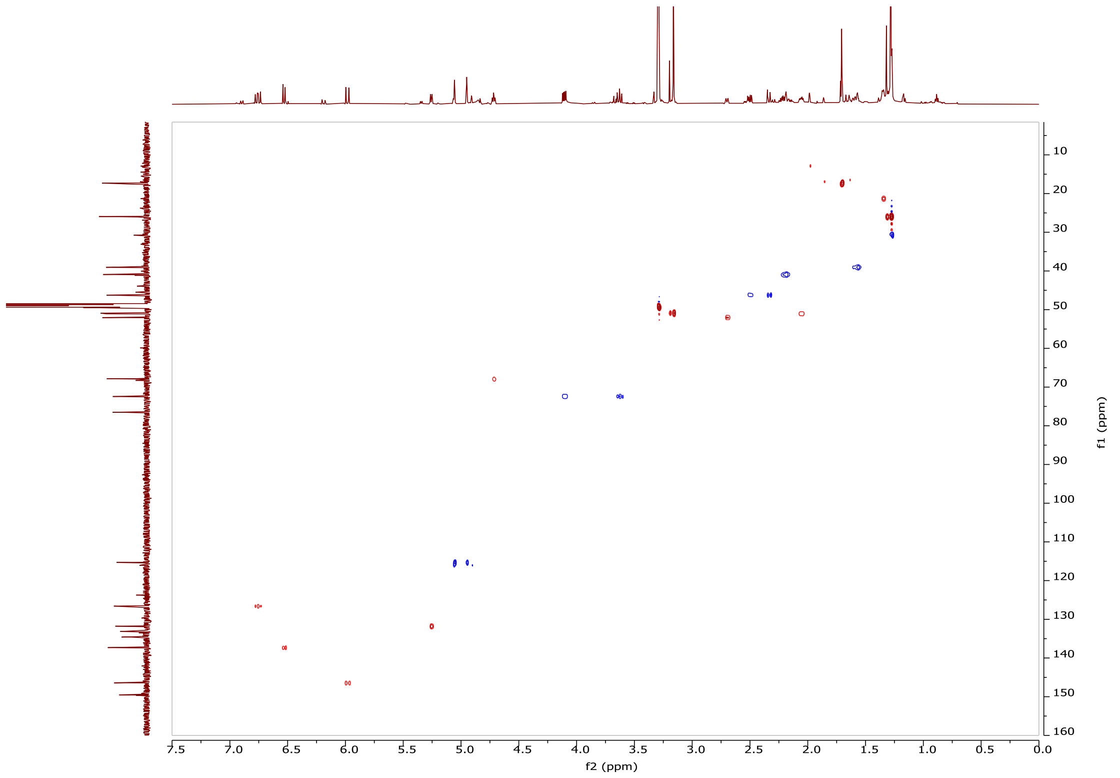

Figure S20. The HSQC spectrum of asterolaurin P (**2**)

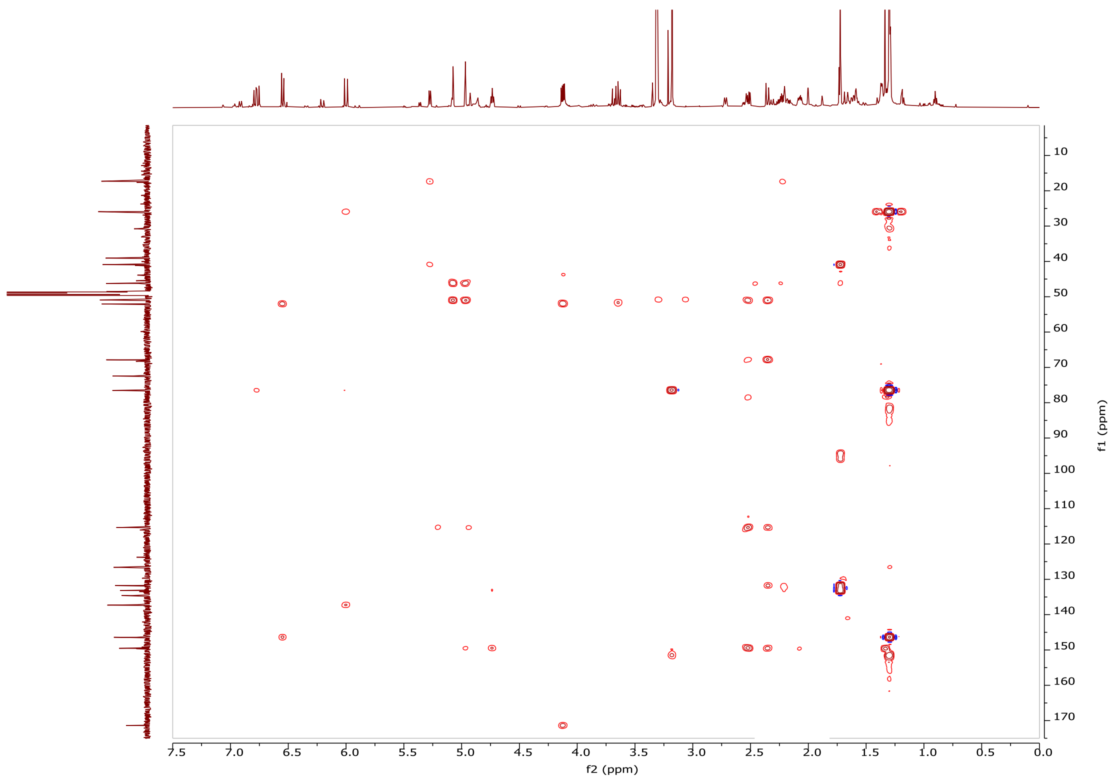

Figure S21. The HMBC spectrum of asterolaurin P (**2**)

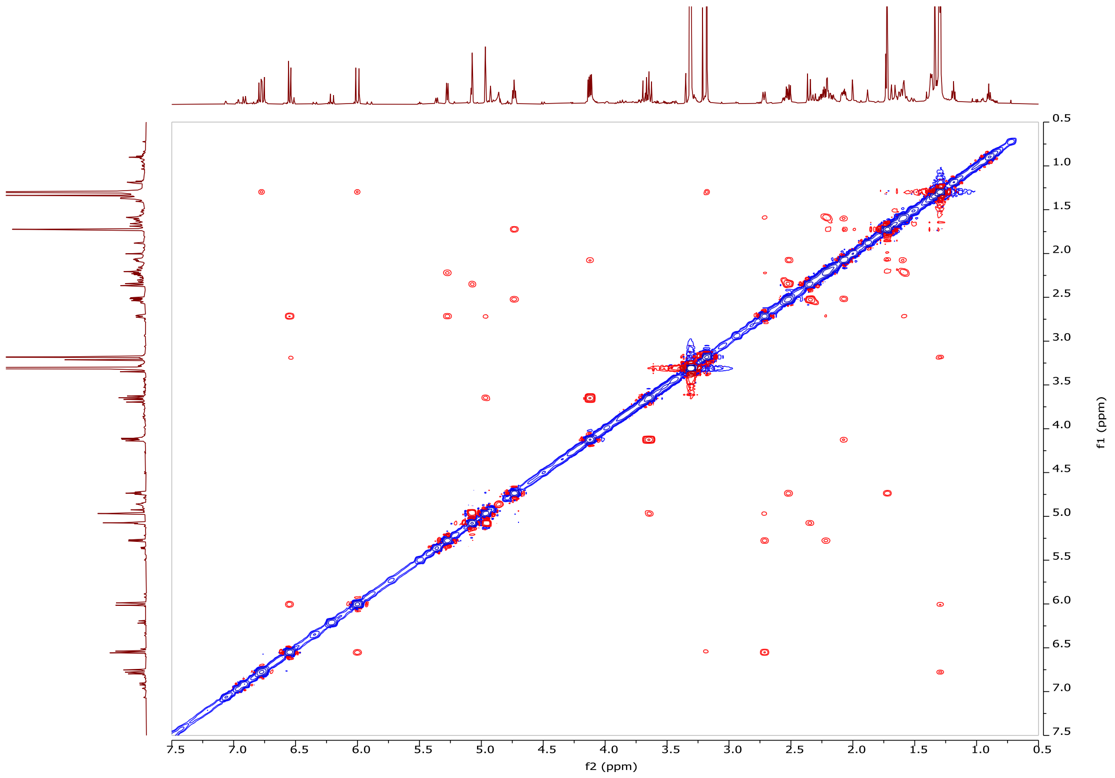

Figure S22. The NOESY spectrum of asterolaurin P (**2**)

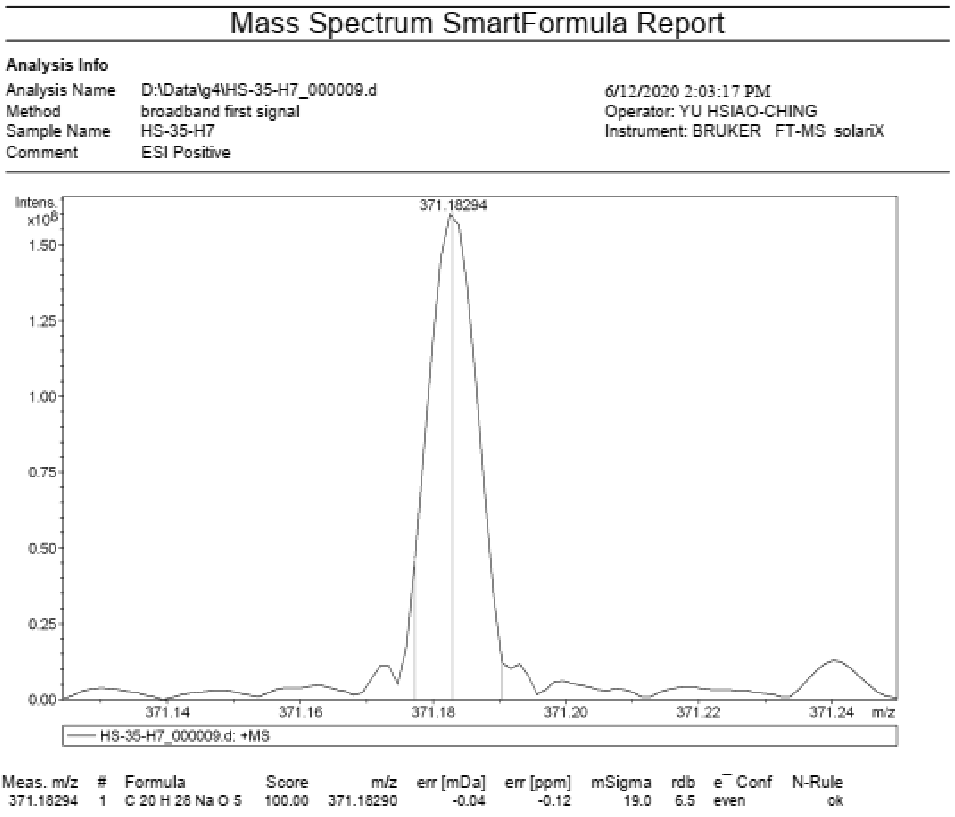

Figure S23. The HRESIMS of asterolaurin Q (**3**)

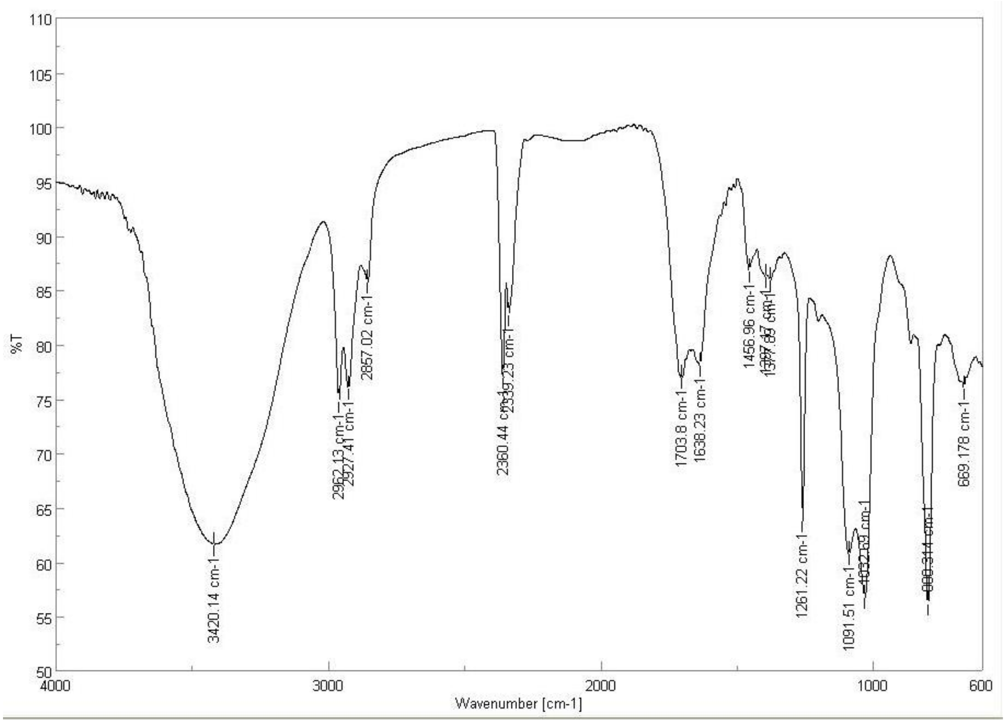

Figure S24. The IR spectrum of asterolaurin Q (**3**)

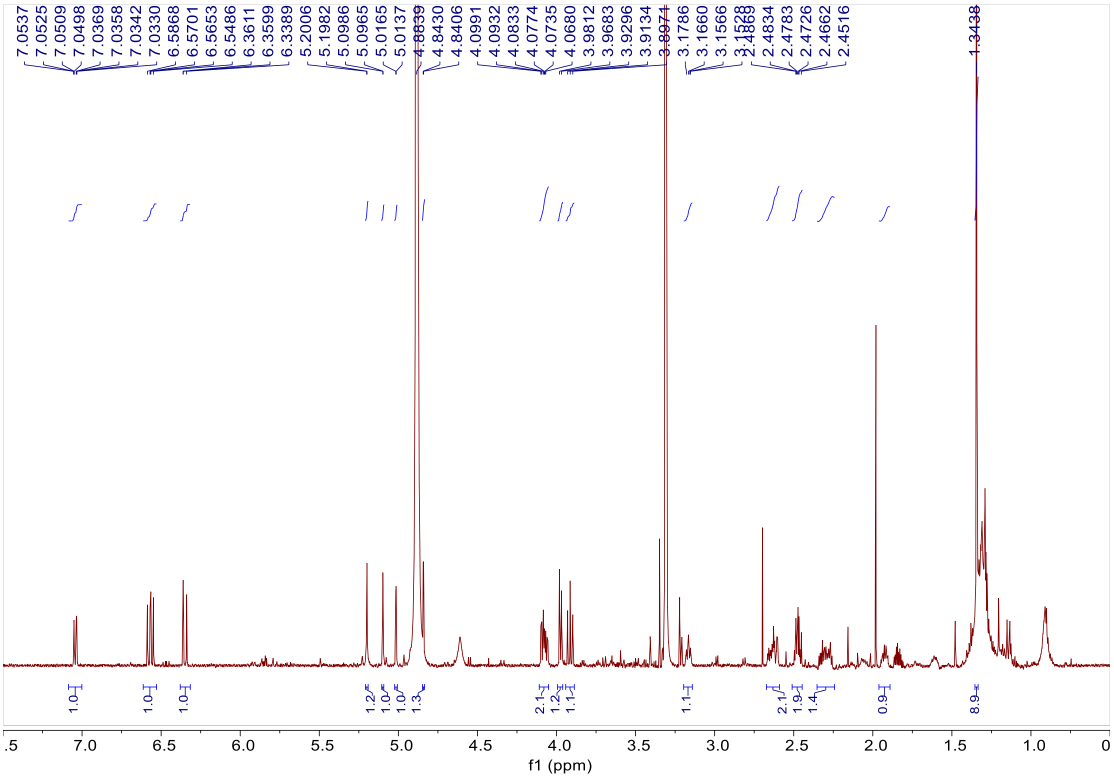

Figure S25. The ^{1}H-NMR spectrum of asterolaurin Q (**3**) (700 MHz in CD_3OD)

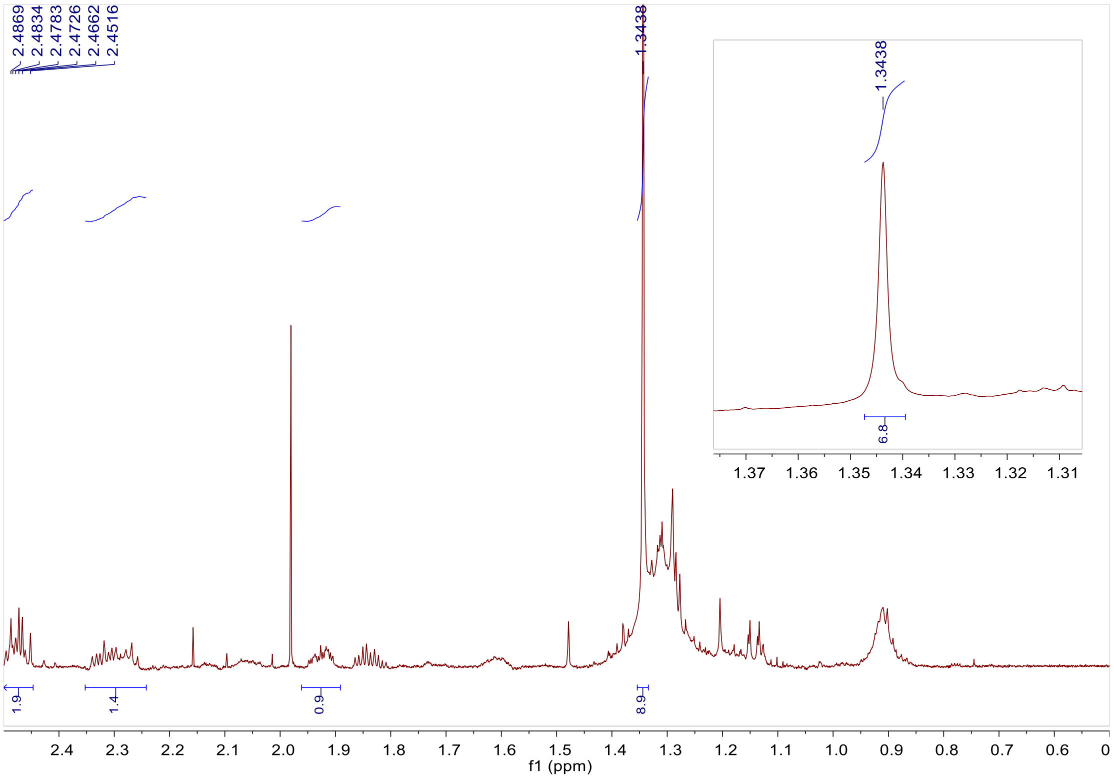

Figure S26. The ^{1}H-NMR spectrum (0.5-2.5 ppm) of asterolaurin Q

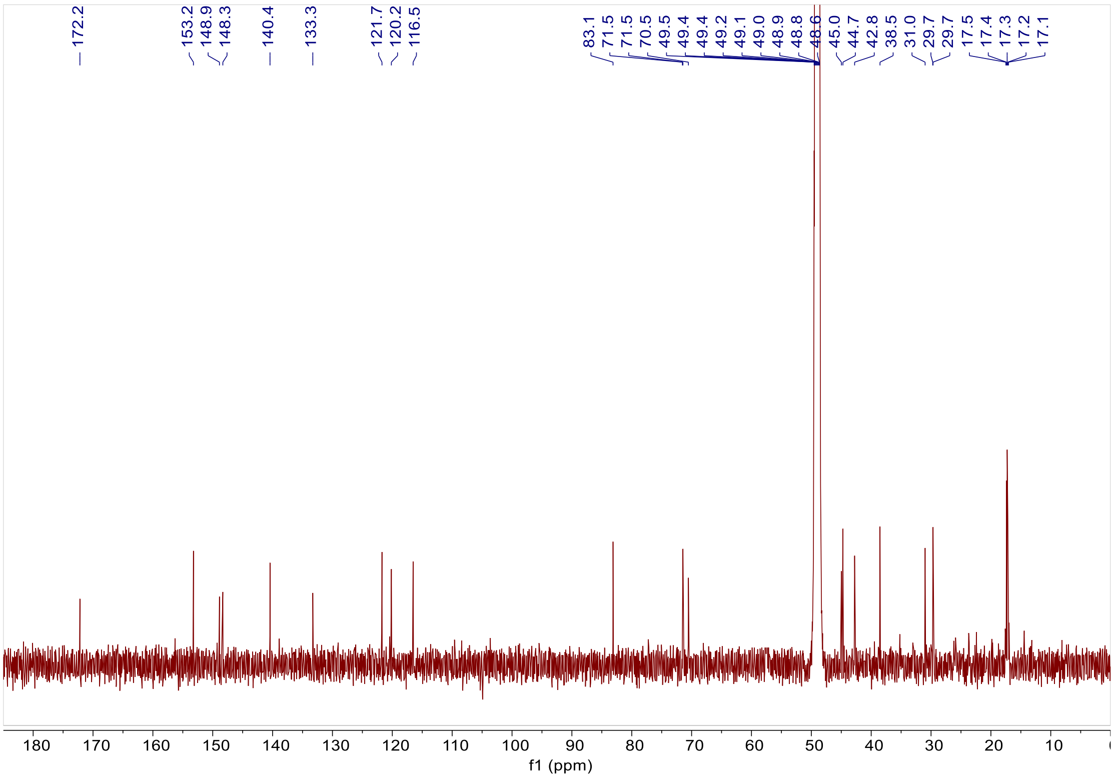

Figure S27. The ^{13}C-NMR spectrum of asterolaurin Q (**3**) (175 MHz in CD_3OD)

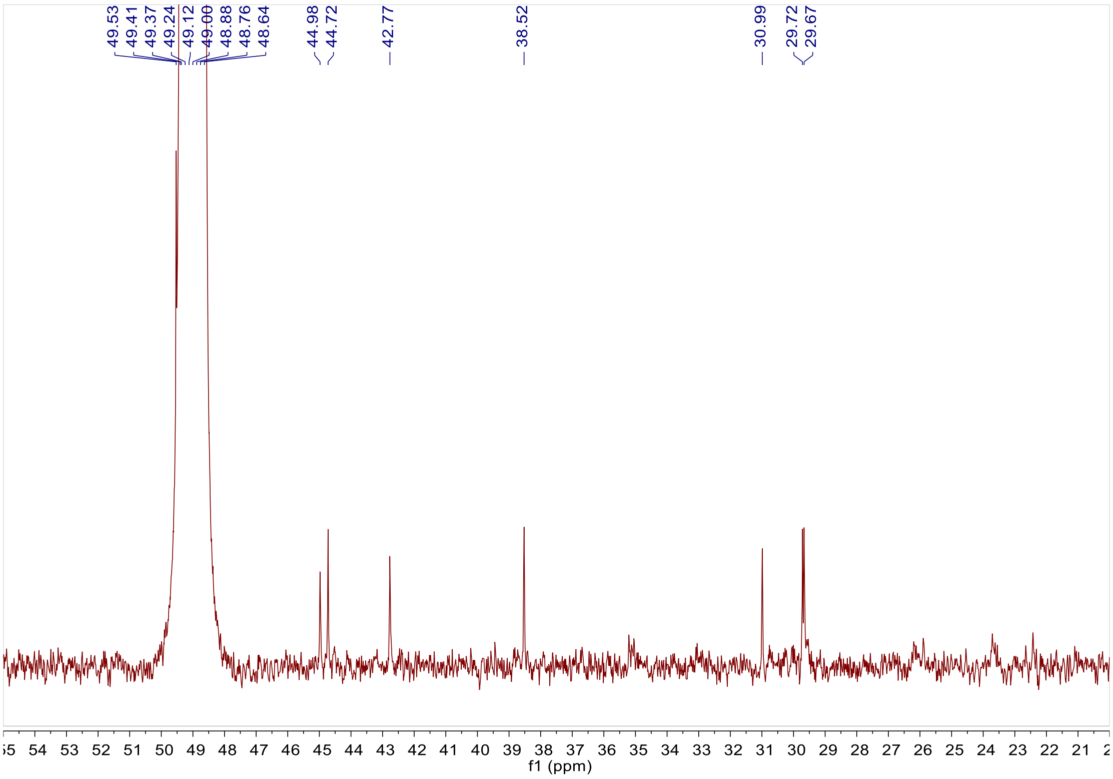

Figure S28. The ^{13}C-NMR spectrum (20-55 ppm) of asterolaurin Q

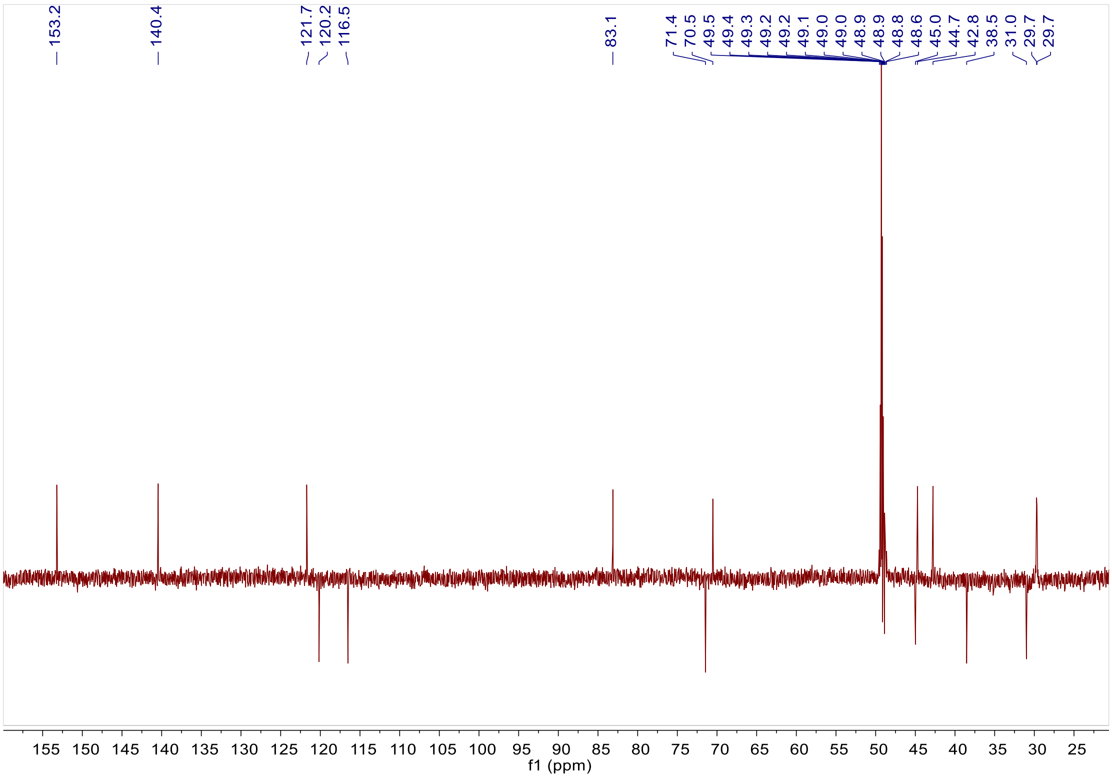

Figure S29. The DEPT spectrum of asterolaurin Q (**3**)

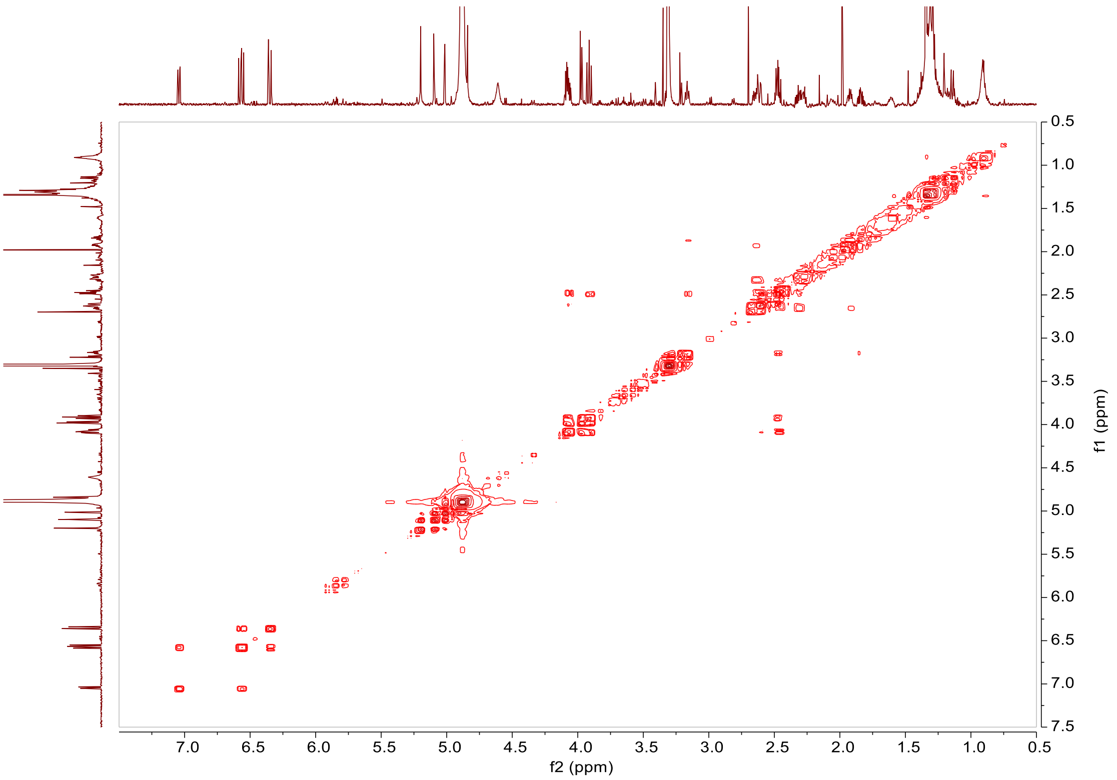

Figure S30. The ^{1}H-^{1}H COSY spectrum of asterolaurin Q (**3**)

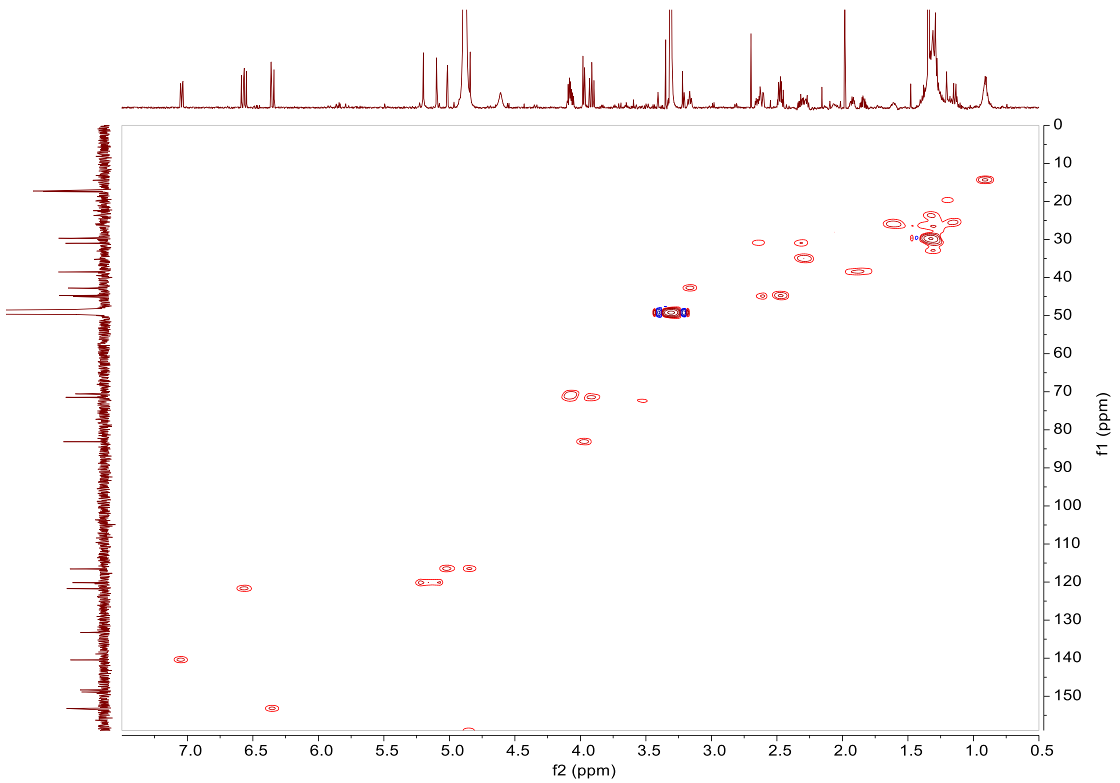

Figure S31. The HSQC spectrum of asterolaurin Q (**3**)

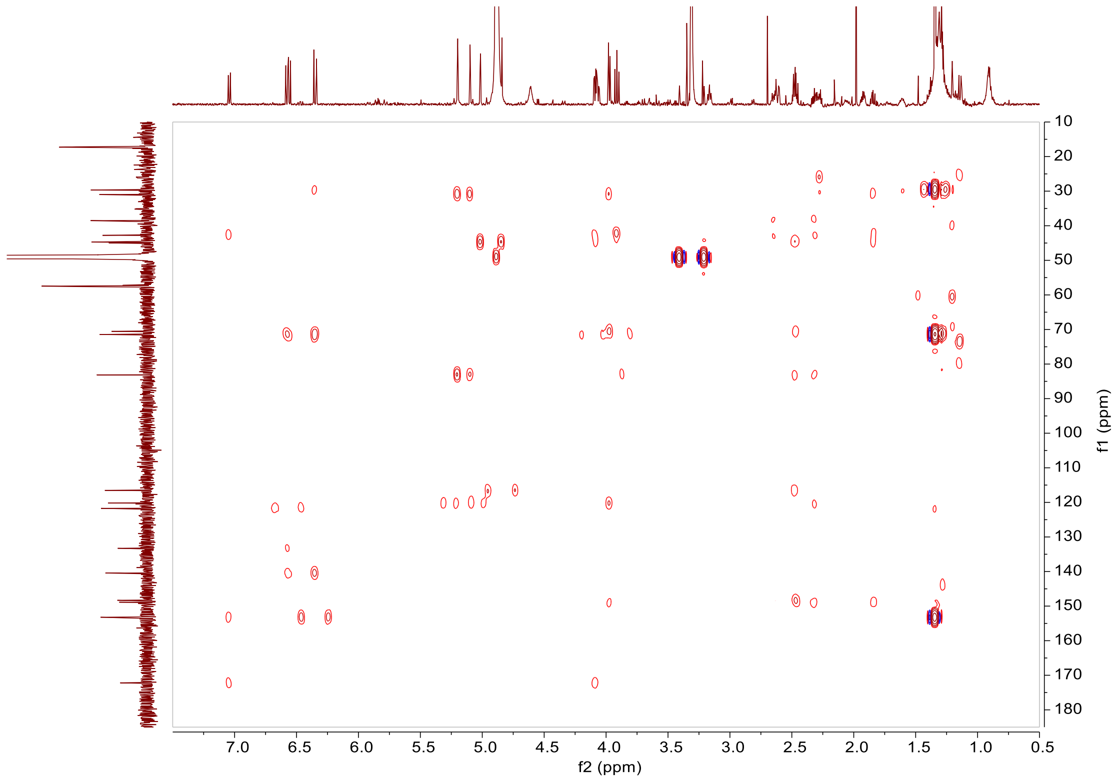

Figure S32. The HMBC spectrum of asterolaurin Q (**3**)

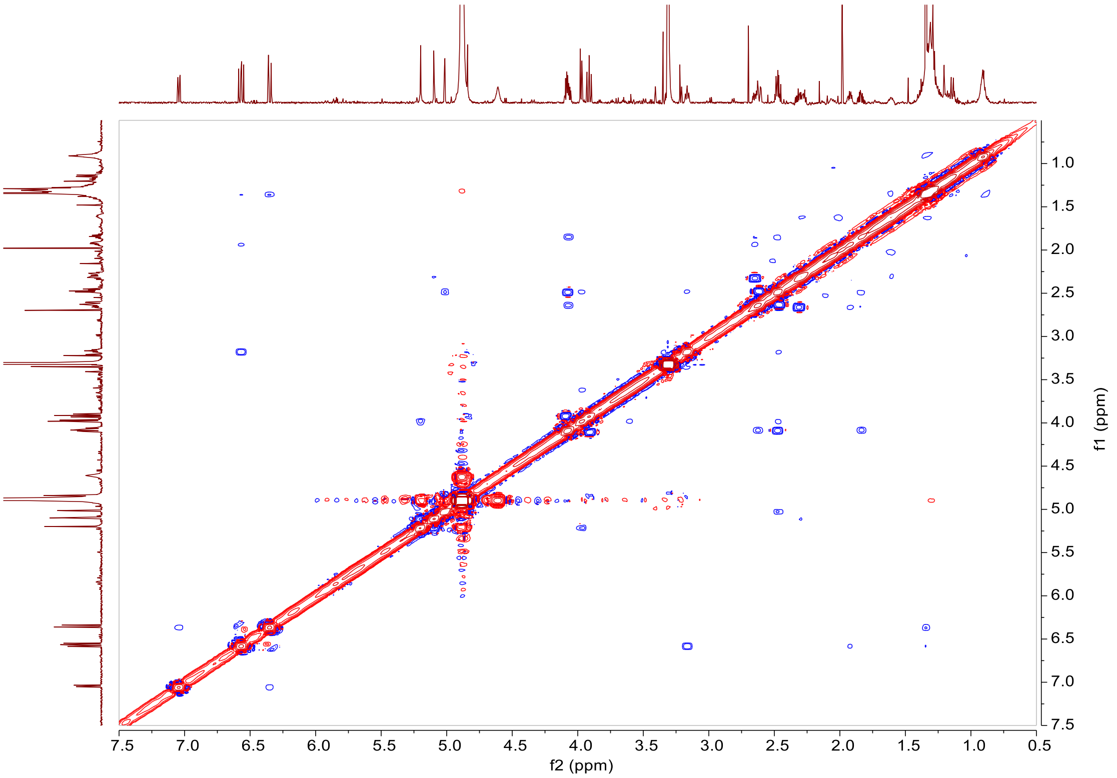

Figure S33. The NOESY spectrum of asterolaurin Q (**3**)

Mass Spectrum SmartFormula Report

Analysis Info

Analysis Name	D:\Data\g4\HS-344-H1_000007.d	6/12/2020 2:41:57 PM
Method	broadband first signal	Operator: YU HSIAO-CHING
Sample Name	HS-344-H1	Instrument: BRUKER FT-MS solariX
Comment	ESI Positive	

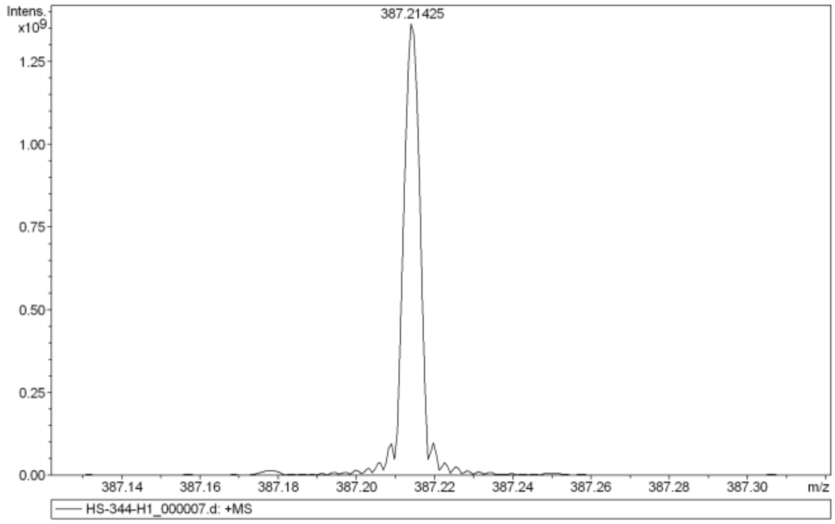

Meas. m/z	#	Formula	Score	m/z	err [mDa]	err [ppm]	mSigma	rdb	e⁻ Conf	N-Rule
387.21425	1	C 21 H 32 Na O 5	100.00	387.21420	-0.06	-0.14	11.6	5.5	even	ok

Figure S34. The HRESIMS of asterolaurin R (**4**)

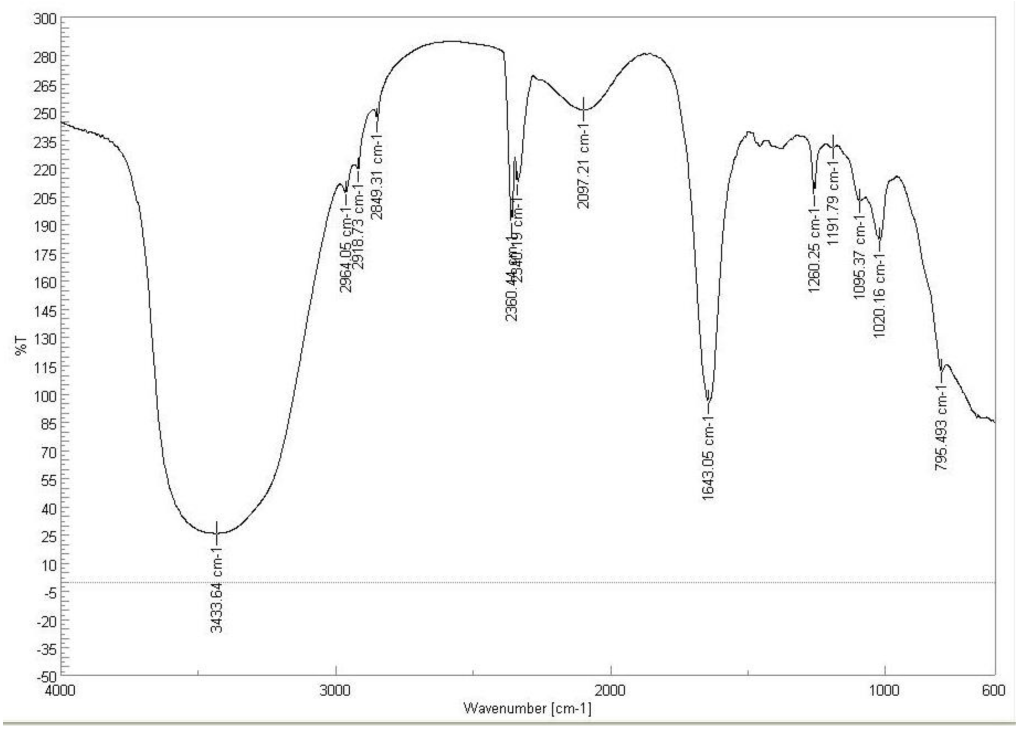

Figure S35. The IR spectrum of asterolaurin R (**4**)

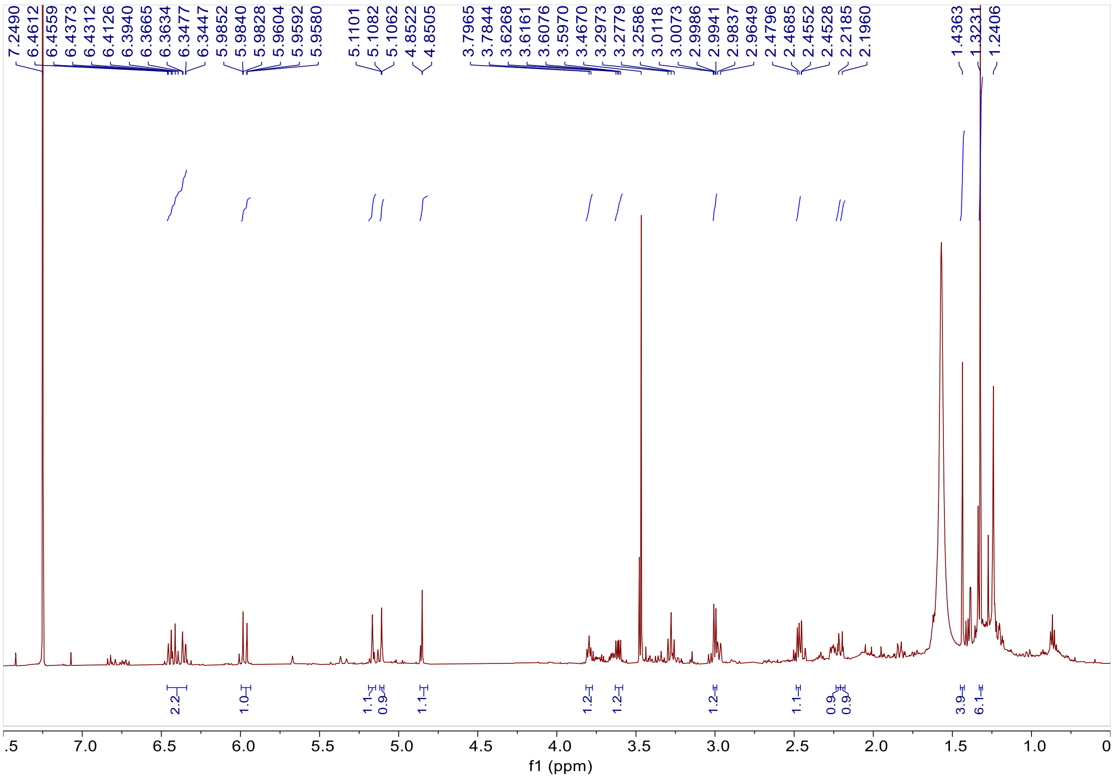

Figure S36. The ^{1}H-NMR spectrum of asterolaurin R (**4**) (600 MHz in $CDCl_3$)

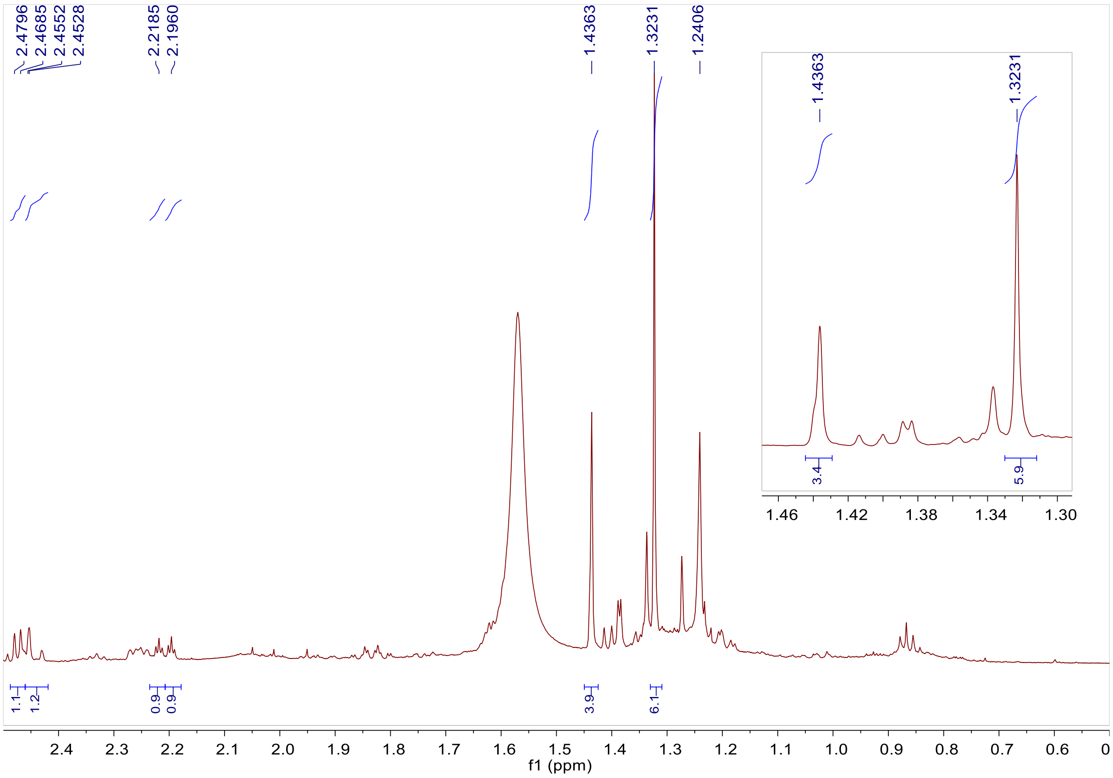

Figure S37. The ^{1}H-NMR spectrum (0.5-2.5 ppm) of asterolaurin R

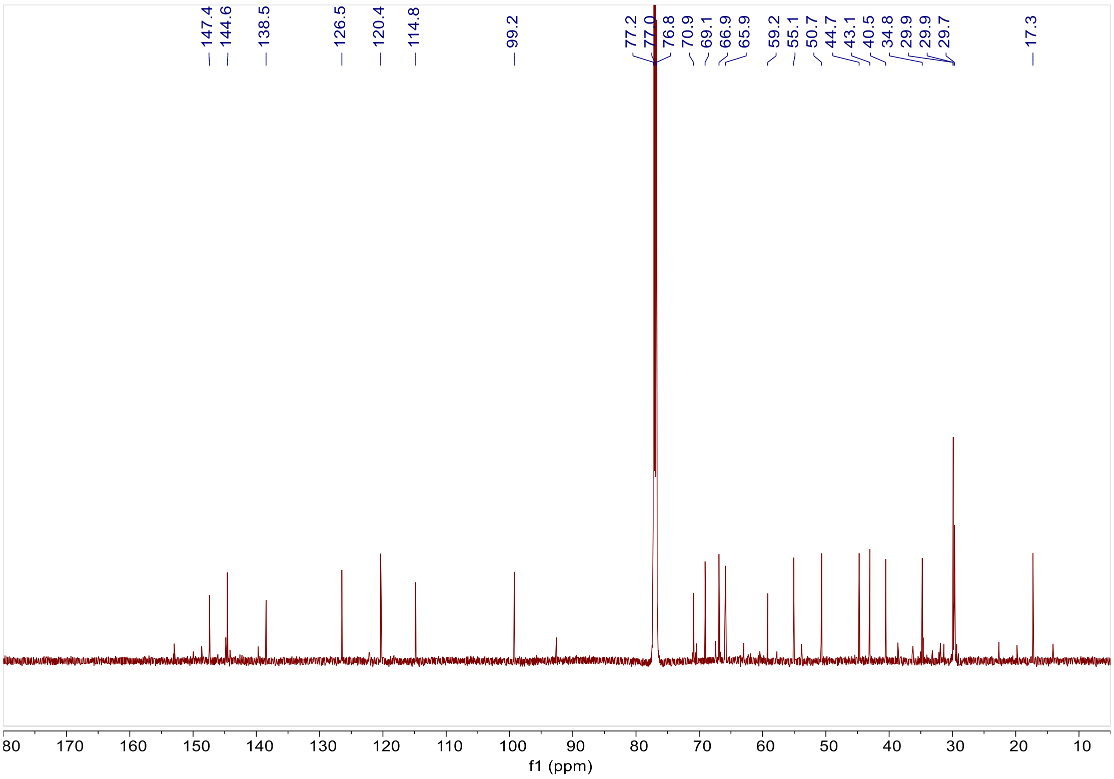

Figure S38. The ^{13}C-NMR spectrum of asterolaurin R (**4**) (150 MHz in $CDCl_3$)

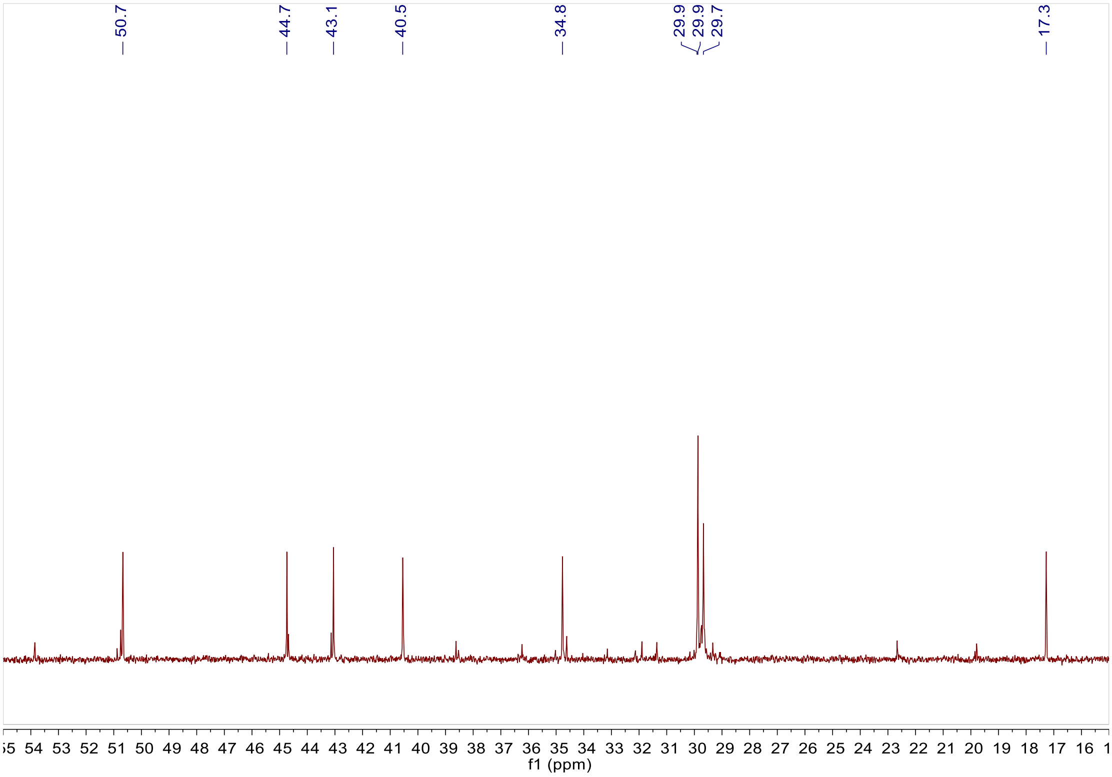

Figure S39. The ^{13}C-NMR spectrum (15-55 ppm) of asterolaurin R

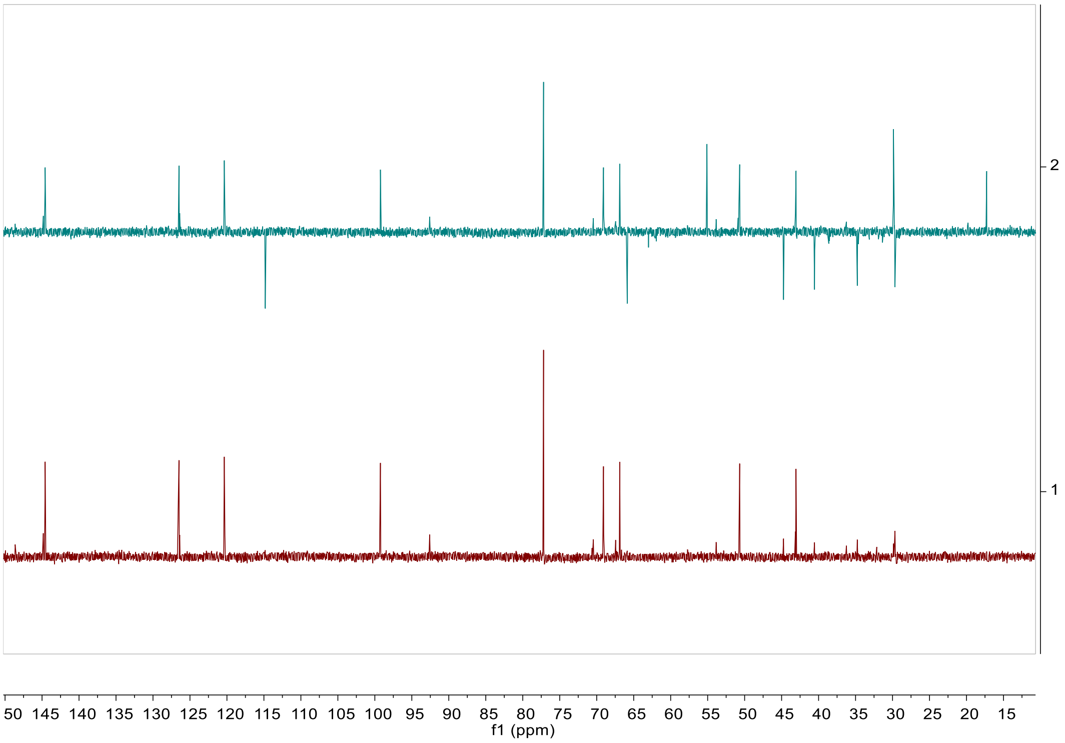

Figure S40. The DEPT spectra of asterolaurin R

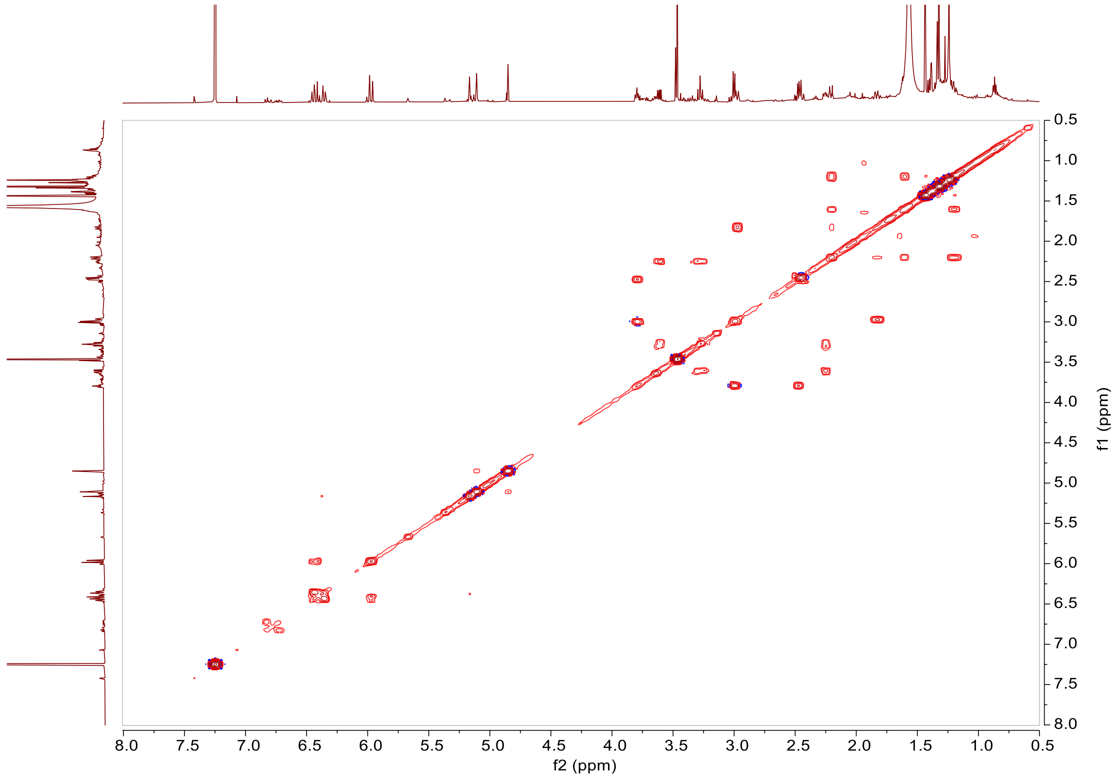

Figure S41. The ^{1}H-^{1}H COSY spectrum of asterolaurin R (**4**)

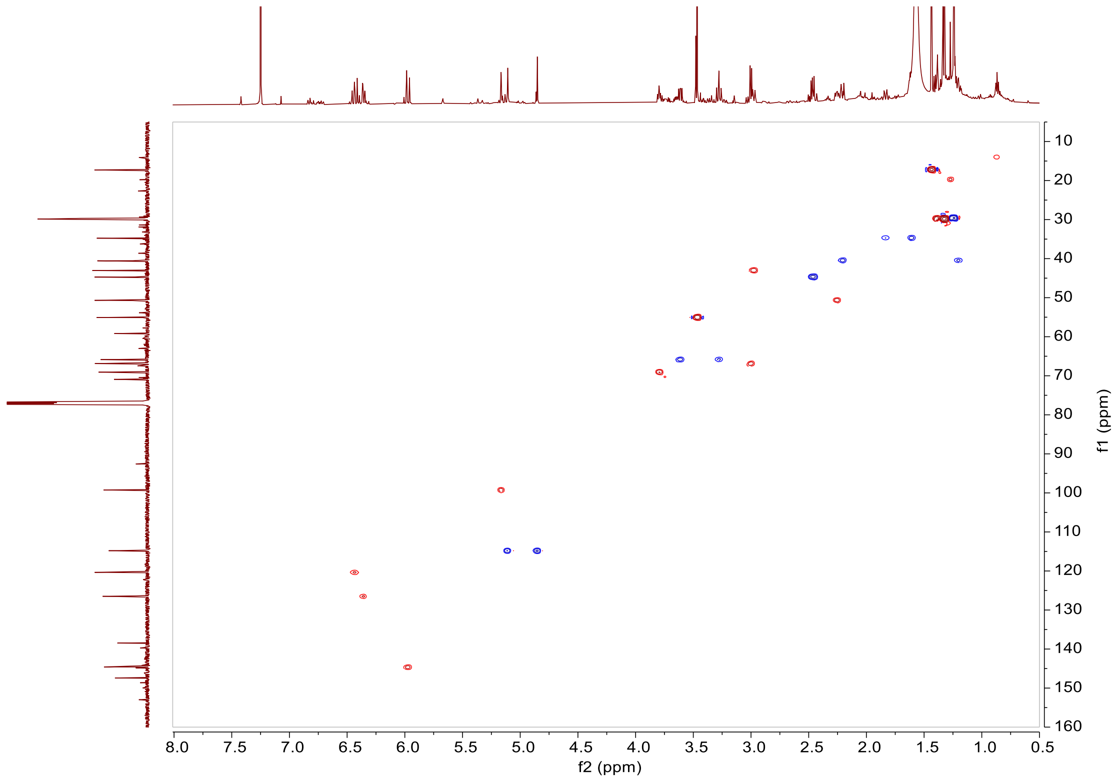

Figure S42. The HSQC spectrum of asterolaurin R (**4**)

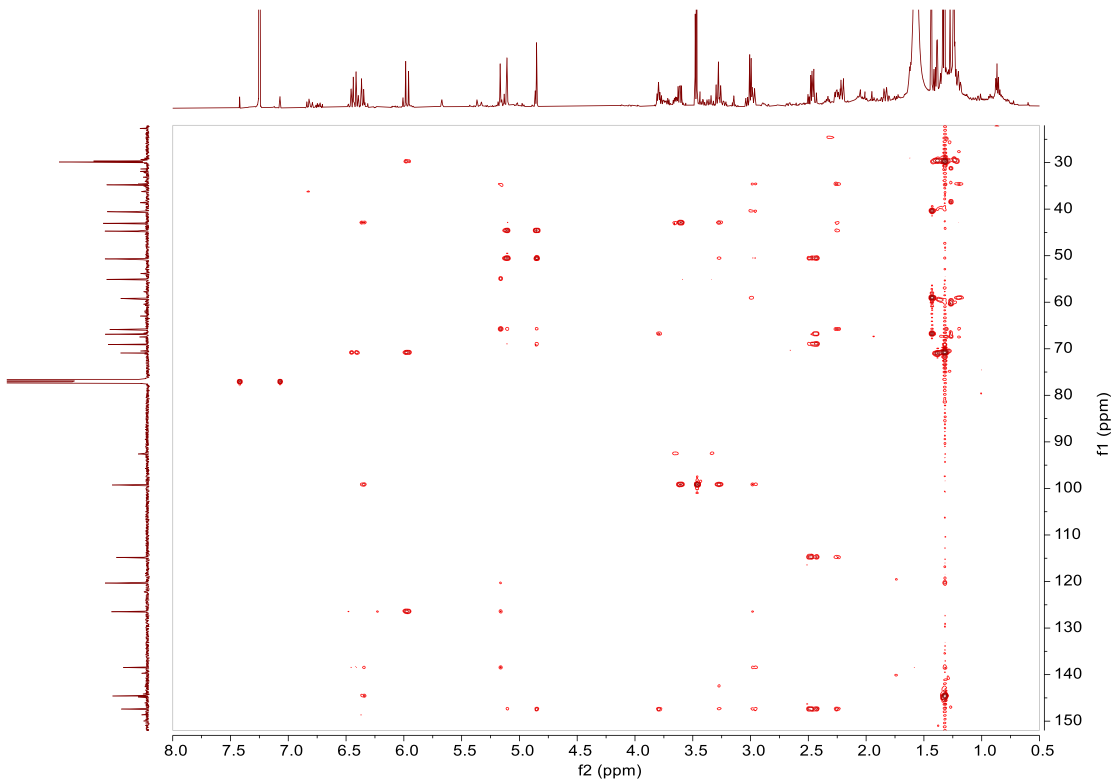

Figure S43. The HMBC spectrum of asterolaurin R (**4**)

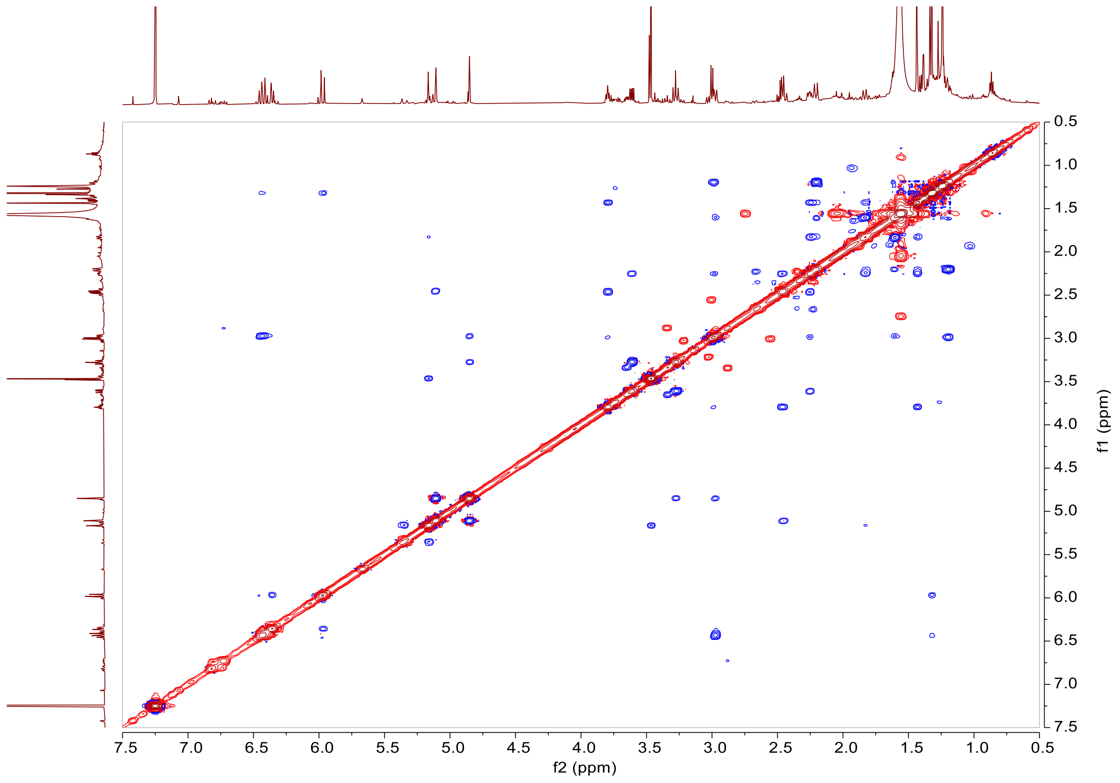

Figure S44. The NOESY spectrum of asterolaurin R (**4**)

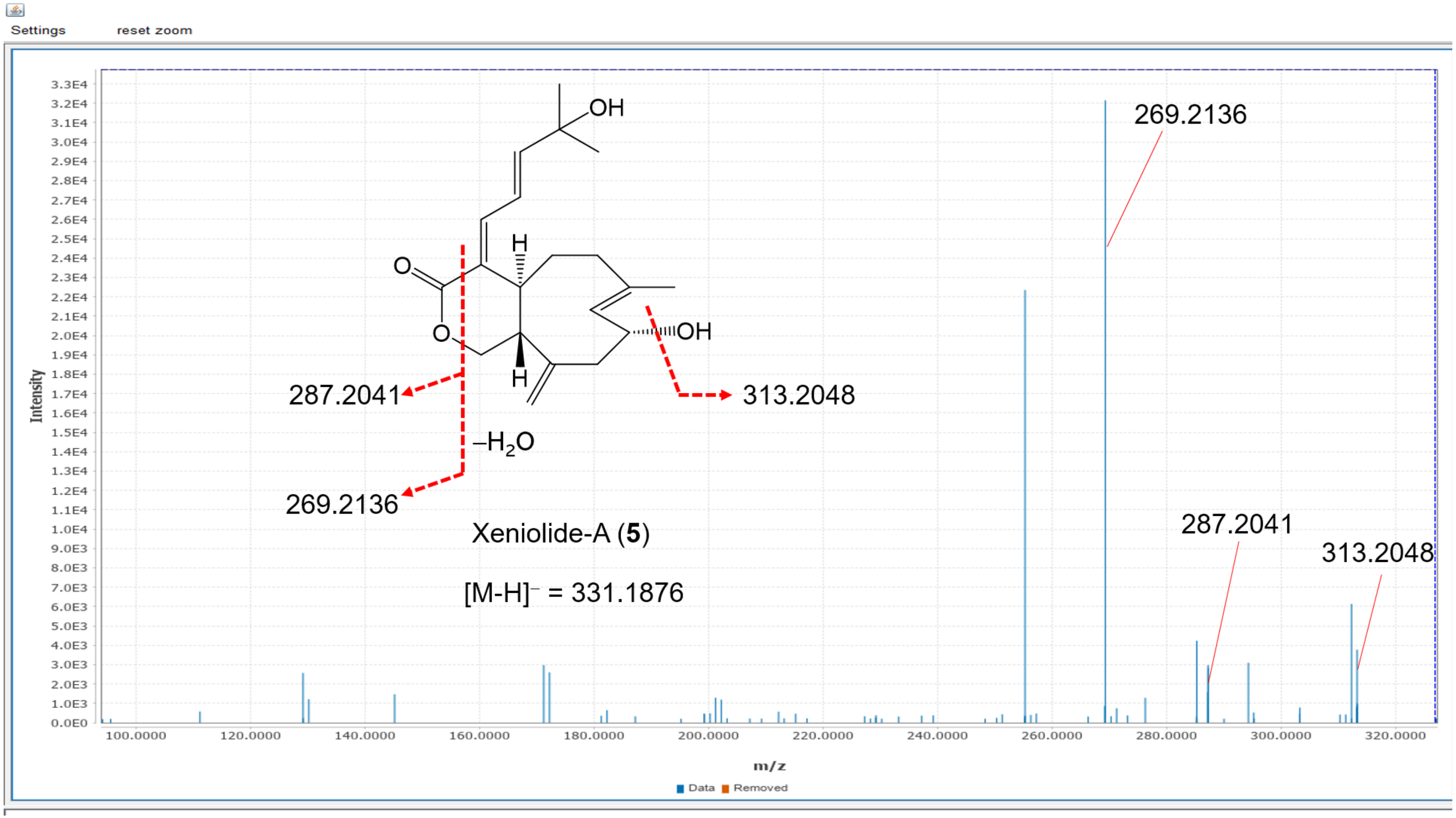

Figure S45. Negative Q-TOF MS/MS spectrum of xeniolide-A (**5**)
